# MiR-22 Deficiency Fosters Hepatocellular Carcinoma Development in Fatty Liver

**DOI:** 10.3390/cells11182860

**Published:** 2022-09-14

**Authors:** Monika Gjorgjieva, Anne-Sophie Ay, Marta Correia de Sousa, Etienne Delangre, Dobrochna Dolicka, Cyril Sobolewski, Christine Maeder, Margot Fournier, Christine Sempoux, Michelangelo Foti

**Affiliations:** 1 Department of Cell Physiology and Metabolism, Faculty of Medicine, University of Geneva, CH-1211 Geneva, Switzerland; 2 Service of Clinical Pathology, Institute of Pathology, Lausanne University Hospital, University of Lausanne, CH-1011 Lausanne, Switzerland; 3 Translational Research Centre in Onco-Haematology, Faculty of Medicine, University of Geneva, CH-1211 Geneva, Switzerland

**Keywords:** non-alcoholic fatty liver disease, hepatocellular carcinoma, microRNA, metabolism, miR-22, Thrombospondin-1

## Abstract

MiR-22 is mostly considered as a hepatic tumor-suppressor microRNA based on in vitro analyses. Yet, whether miR-22 exerts a tumor-suppressive function in the liver has not been investigated in vivo. Herein, in silico analyses of miR-22 expression were performed in hepatocellular carcinomas from human patient cohorts and different mouse models. Diethylnitrosamine-induced hepatocellular carcinomas were then investigated in lean and diet-induced obese miR-22-deficient mice. The proteome of liver tissues from miR-22-deficient mice prior to hepatocellular carcinoma development was further analyzed to uncover miR-22 regulated factors that impact hepatocarcinogenesis with miR-22 deficiency. MiR-22 downregulation was consistently observed in hepatocellular carcinomas from all human cohorts and mouse models investigated. The time of appearance of the first tumors was decreased and the number of tumoral foci induced by diethylnitrosamine was significantly increased by miR-22-deficiency in vivo, two features which were further drastically exacerbated with diet-induced obesity. At the molecular level, we provide evidence that the loss of miR-22 significantly affects the energetic metabolism and mitochondrial functions of hepatocytes, and the expression of tumor-promoting factors such as thrombospondin-1. Our study demonstrates that miR-22 acts as a hepatic tumor suppressor in vivo by restraining pro-carcinogenic metabolic deregulations through pleiotropic mechanisms and the overexpression of relevant oncogenes.

## 1. Introduction

Liver cancer is the sixth most frequent human cancer and the fourth leading cause of mortality among all cancer types [1,2]. Hepatocellular carcinoma (HCC) is the most common primary liver cancer, representing 75% of all cases [1,2]. Hepatic viral infections (HBV, HCV), alcohol abuse and obesity/non-alcoholic fatty liver disease (NAFLD) represent the three main etiological factors associated with HCC. The global prevalence of obesity/NAFLD is expected to further increase in the future and to become a major cause of HCC development [3]. The dogmatic view of NAFLD with HCC as an end stage describes a hepatic pathology unfolding in a linear manner, with the first stage of NAFLD being simple steatosis that can further progress to non-alcoholic steatohepatitis (NASH), fibrosis, and finally cirrhosis, a major risk factor for HCC [3,4,5]. However, it is now clear that HCC can also arise from NASH in the absence of cirrhosis [3,4,5]. In this context, in addition to inflammation, chronic metabolic disorders and stress-activated pathways, commonly associated with NAFLD/NASH, are also important drivers of tumor initiation and progression. These include exacerbated glycolysis, gluconeogenesis, lactate synthesis, inhibition of mitochondrial activity, and activation of pathways that favor cell proliferation, such as insulin growth factor-stimulated signaling [6,7]. Understanding more precisely the molecular mechanisms of metabolic disorders associated with NAFLD/NASH that contribute synergistically with inflammation to tumorigenesis should help to develop relevant therapeutic strategies for these patients.

HCCs are characterized by an important heterogeneity and a high number of mutations, of which the most frequent are found in the promoter of *TERT* and the *CTNNB1* and *TP53* genes [8]. While identification of major HCC mutations provided important insights for patient survival and disease outcome, these mutations are poorly druggable for therapeutic purposes. Importantly, other molecular mechanisms deregulated with NAFLD/NASH can lead to alterations in the expression or activity of cancer-related factors that mimic mutations in tumor suppressors or oncogenes. A well-known example is the deregulation of PTEN, a very potent and haplo-insufficient tumor suppressor [9,10], whose expression/activity is strongly downregulated in the liver with steatosis [11,12]. Non-genomic alterations of cancer-promoting factors with NAFLD/NASH may occur through various mechanisms including epigenetic changes [13,14], deregulation of small/long non-coding RNAs [15,16,17], or alterations of the expression/activity of other regulatory factors, such as RNA-binding proteins [18,19]. In this regard, microRNAs (miRNAs) have been repeatedly described as important regulators of hepatic metabolism and carcinogenesis, and the expression/activity of specific miRNAs is drastically affected in NAFLD/NASH and HCC [16]. A major feature of miRNAs is that one single miRNA has the potential to simultaneously downregulate hundreds of target genes, thus impacting whole gene networks and various cellular processes. This miRNA characteristic is one of the most relevant and prominent arguments to consider these small regulatory non-coding RNAs as efficient therapeutic targets for liver diseases and cancers [20,21]. Confirming this hypothesis, therapeutic compounds targeting miR-122 and miR-34 in the liver have entered clinical trials as potential drugs for HCV infection and liver cancer, respectively [22,23].

We have recently described the important role of microRNA-22 (miR-22) in obesity development and the regulation of hepatic metabolism [24]. Genetic ablation of miR-22 in mice (miR-22KO mice) was asymptomatic in normal breeding conditions, whereas it led to an exacerbation of hepatic steatosis and obesity development in mice submitted to a highly caloric (high-fat content) obesogenic diet. In the same study, our proteomic analysis of miR-22-depleted hepatic mouse tissues revealed an increased expression of key actors of glycolysis and lipid synthesis, indicating that miR-22 broadly impacts hepatic cellular metabolism. Since we demonstrated that miR-22 deficiency strongly promotes NAFLD and obesity, the question arises whether deregulations of miR-22 expression/activity may also contribute to hepatic cancer development. In agreement with this hypothesis, several studies reported miR-22 downregulation in HCC, using tumoral and non-tumoral tissues of patients [25,26,27,28], and in the serum or blood cells of HCC patients [29,30], therefore suggesting a tumor-suppressive role for this miRNA in the liver. Further supporting a tumor-suppressor role for miR-22, oncogenes such as galectin-1 (*LGALS1*) [31], ezrin (*EZR*) [32], histone deacetylase 4 (*HDAC4*) [33] tyrosine 3-monooxygenase/tryptophan 5-monooxygenase (*YWHAZ*) [28], specificity protein 1 (*SP1*) [34], and basigin (*CD147*) [27] were reported to be hepatic targets of miR-22. However, conclusions about the role of miR-22 in liver cancer are based on correlative expressions of miR-22 and its targets in HCC biopsies of the above studies, or on studies using xenograft HCC models that poorly represent the orthotopic development of HCC in a NAFLD context. An oncogenic role of miR-22 was also suggested, for example, in HBV-related HCC, prostate cancer, or chronic lymphocytic leukemia (CLL), where miR-22 upregulation was shown to target important tumor suppressors such as PTEN or ERα [35,36,37]. It is therefore currently unclear whether miR-22 exerts a tumor-suppressive or oncogenic role in HCC initiation and progression in vivo.

In this study, we confirmed the downregulation of miR-22 in multiple human cohorts of HCC patients and various mouse models of this disease. HCC development in the liver of lean and obese mice was further assessed in wild-type (WT) and miR-22 genetically deficient mice. Finally, metabolic pathways and cellular targets of miR-22, which potentially contribute to hepatic cancer development, were uncovered by proteomic analyses.

## 2. Materials and Methods

### 2.1. Mouse Studies

#### 2.1.1. Mouse Generation and Housing

Male mice bearing a total constitutive genetic ablation of MiR22 (miR-22KO mice) were obtained as previously described [24]. WT littermate mice were used as controls in all mouse experiments. MiR22 deletion was confirmed by conventional PCR-based genotyping and RT-qPCR. All groups were housed at the animal facility of University of Geneva. Food and water access was ad libitum. Mice were housed in groups of 3–6 mice per cage, following a 12 h dark/light cycle. Each cage contained enrichment (e.g., cardboard house, cotton for nesting). Breeding, housing, and experimentation protocols were approved by the Geneva Health Head office (authorization numbers GE-10-19 and GE-11-17) and were in accordance with the Swiss guidelines for animal experimentation.

#### 2.1.2. Hepatic Tumor Induction

Diethylnitrosamine (DEN, 25 mg/kg of body weight, Sigma-Aldrich, St. Louis, MO, USA) was administered by intraperitoneal injection in 15-day-old pups of miR-22KO mice and WT littermates to induce hepatic cancer development.

#### 2.1.3. Diet-Induced Steatosis and Obesity in DEN-Treated Mice

WT littermate mice and miR22KO mice treated with DEN were fed standard chow diet and water ad libitum until the age of two months. Mice of each strain were then randomly organized in two groups and submitted to a high-fat diet (HFD: 60 kJ% fat, ssniff^®^ EF acc. D12492 (II) mod., Soest, Germany) or standard chow diet (CD: 11 kJ% fat, ssniff^®^ EF R/M control, Soest, Germany), matched for its composition with the HFD, until sacrifice at 11 months of age (9 months of diet). The total number of mice in each cohort was 14 for WT mice fed CD, 14 for WT mice fed HFD, 7 for miR22KO mice fed CD, and 9 for miR22KO mice fed HFD.

#### 2.1.4. Computer Tomography (CT) Analysis

Mouse livers were imaged at sacrifice, and every month (starting from 5 months up to 11 months of age) for a subset of mice, via computer tomography (CT) scan—Quantum GX microCT software (PerkinElmer, Waltham, MA, USA). Contrast enhancement was obtained by retro-orbital injection of 100 μL of ExiTron nano6000 (Viscover, Berlin, Germany). CT scans were then analyzed using the open-source Horos Software (v3.3.6, Horos Project). The 3D representation of tumors was obtained using the same software.

#### 2.1.5. Glucose Tolerance Test

The glucose tolerance test (GTT) was performed as previously described [24]. Briefly, 3–5 mice from each group at 5 months of age were starved overnight and a glucose load of 1.5 g/kg was administered via intraperitoneal injection. Glycemia was measured at indicated times (0, 15, 30, 60, 90 and 120 min) using Glucotrend Active (Roche, Basel, Switzerland).

#### 2.1.6. Plasma Analysis

Blood collected during sacrifice was centrifuged and the plasma was analyzed via the Cobas 8000 system (Roche, Basel, Switzerland).

### 2.2. Cell Culture

#### 2.2.1. Cell Maintenance

HepG2 and Huh7 cells (human hepatoma) were purchased at the ATCC (Manassas, VA, USA). They were cultured in DMEM medium (1 g/L glucose, Thermo Fischer Scientific, Waltham, MA, USA), supplemented with 10% fetal bovine serum (FBS, Thermo Fischer Scientific, Waltham, MA, USA) and 1% penicillin-streptomycin (PS, Thermo Fisher Scientific, Waltham, MA, USA). AML12 cells were cultured in DMEM/F12 medium supplemented with 100nM of dexamethasone (Sigma Aldrich, St. Louis, USA), 1% of insulin-transferrin-selenium (Thermo Fisher Scientific, Waltham, MA, USA), 10% FBS (Thermo Fisher Scientific, Waltham, MA, USA), and 1% PS (Thermo Fisher Scientific, Waltham, MA, USA).

#### 2.2.2. Cell Transfection

Cells were transfected using 6 µL interferin (Polyplus transfection, Illkirch, France) and 200 µL optimem (Thermo Fisher Scientific, Waltham, MA, USA) 24 h post-seeding (6-well plates), following manufacturer’s instructions. For miRNA overexpression, cells were transfected with miRIDIAN microRNA miR-22-3p mimic or miRIDIAN microRNA Mimic Negative Control #1 (Horizon Discovery, Cambridge, UK) with concentrations ranging between 1 and 20 nM depending on the assay. For gene silencing, 10 nM of control siRNA (AllStars Negative Control siRNA, Qiagen, Hilden, Germany) or siRNA against thrombospondin 1 (siTSP1, Hs_THBS1_3 FlexiTube siRNA, Qiagen, Hilden, Germany) was transfected, also using interferin and optimem, following manufacturer’s instructions.

#### 2.2.3. Cell Migration Assay (Boyden Chamber Assay)

HepG2 cells were seeded in a 6-well plate (200,000 cells per well) and transfected as described in Section 2.2.2. Boyden Chamber’s assay was performed as previously described [11]. The assay was performed 4 times (hexaplicates per condition).

#### 2.2.4. Cell Proliferation

HepG2 and Huh7 cells were seeded in a 6-well plate (200,000 and 100,000 cells per well, respectively) and transfected as described in Section 2.2.2. Proliferation was assessed at 48 and 72 h after transfection under steatotic conditions (cells treated with 150 μM oleate/palmitate and 4.5 g/L of glucose in the medium). After trypsinization, cells were counted with a Neubauer cell. The assay was performed 3 times (quadruplicates per condition).

#### 2.2.5. Luciferase-Based Gene Reporter Assay

HepG2 cells were seeded in a 6-well plate (200,000 cells per well) and 24 h after seeding they were transfected with 1nM of miRIDIAN microRNA miR-22-3p mimic or miRIDIAN microRNA Mimic Negative Control #1 (Horizon Discovery, Cambridge, UK), together with 600 ng/μL of a luciferase-reporter vector (LightSwitch 3′-UTR Reporter Go Clone vector for human TSP1; ref. S811182, vector name: pLightSwitch-3UTR-TSP1, Active Motif, Carlsbad, CA, USA). The vector encodes a hybrid transcript that contains the luciferase coding sequence fused to the 3′-UTR sequence of human TSP1. Transfection of the oligonucleotides and the vector was performed using Viromer Red (Lipocalyx, Halle, Germany), as described in the user manual. At 48 h after transfection, cells were washed with PBS 1×, scraped and lysed in 100 µL of PBS 1×. Luciferase assays were then performed in each sample using LightSwitch Luciferase Assay Kit (Active Motif, Carlsbad, CA, USA), as described in the user manual, and the luminescence in each sample was determined by the SpectraMax Paradigm (Molecular Devices, Winnersh, UK). The luminescence value for each assay was further reported to the protein concentration of the sample for normalization. Protein content was determined using the Pierce BCA Protein Assay Kit (Thermo Scientific, Waltham, MA, USA).

To determine the functional relevance of the 3 different predicted miR-22 binding sites on the 3′-UTR of TSP1 (based on the miRWalk v2.0 prediction database), each of these sites was mutated in the pLightSwitch-3UTR-TSP1 vector, using QuikChange Multi Site-Directed Mutagenesis Kit (Agilent, Santa Clara, CA, USA) with the primers indicated in the Supplementary Figure S6. The Luciferase assay was then performed with the vector carrying each specific mutation (Mut1, Mut2 or Mut3), in the same way as the vector with the wild-type sequence.

### 2.3. Mitochondrial Respiration and Glycolysis Analyses

#### 2.3.1. Long-Chain Fatty acid Oxidation Stress Test

Huh7 and AML12 cells were seeded in a 6-well plate (350,000 cells per well) and transfected as described in Section 2.2.2. At 24 h after transfection, cells were replated in a 96-well Seahorse Agilent plate at 40,000 cells per well. At 24 h after the replating, Seahorse XF Long Chain Fatty Acid Oxidation Stress Test (103672-100 kit, Agilent, Santa Clara, CA, USA) was performed precisely as described by the Seahorse Agilent user guide (*n* = 3, in triplicate). The concentrations of the drugs used were as follows: etomoxir—4 μM, oligomycin—1.5 μM, FCCP—2 μM, and rotenone/antimycin A—0.5 μM. The assay was performed using the Seahorse XFe96 Analyzer with the standard template for the protocol for Seahorse XF Substrate Oxidation Stress Test. After running the assay, Hoechst 33,342 (1 µg/mL final concentration) was added to the medium and the cell number in each well was determined using a Cytation 5 cell imaging multimode reader (BioTek, software Gen5™, Agilent, Santa Clara, CA, USA). Data from the Seahorse analyzer were then normalized to cell number and represented as described in the Seahorse Agilent user guide.

#### 2.3.2. Glycolytic Rate Assay (GlycoRate)

Huh7 and AML12 cells were seeded in a 6-well plate (350,000 cells per well) and transfected as described in Section 2.2.2. At 24 h after transfection, cells were replated in a 96-well Seahorse Agilent plate at 40,000 cells per well. At 24 h after the replating, Seahorse XF Glycolytic rate assay (GlycoRate 103344-100 kit, Agilent, Santa Clara, CA, USA) was performed precisely as described by the Seahorse Agilent user guide (*n* = 3, in triplicate). The Glycolytic Rate Assay allows to evaluate the basal rate of glycolysis in the cells. In this assay, the injection of 2-deoxy-D-glucose (2-DG) serves as a control in which the measured medium acidification is a consequence of glycolysis, considering that this compound competes with glucose and diffuses through the plasma membrane in a similar fashion, but it is not metabolized. The concentrations of the drugs used were as follows: rotenone/antimycin A—0.5 μM, 2-DG—50 mM. The assay was performed using the Seahorse XFe96 Analyzer with the standard template for the protocol for Seahorse XF Glycolytic rate assay. After running the assay, Hoechst 33,342 (1 µg/mL final concentration) was added to the medium and the cell number in each well was determined using a Cytation 5 cell imaging multimode reader (BioTek, software Gen5™, Agilent, Santa Clara, CA, USA). Data from the Seahorse analyzer were then normalized to cell number and represented as described in the Seahorse Agilent user guide.

### 2.4. RT-qPCR Analysis

Flash frozen mouse tissues and cells were suspended in 1mL of Trizol (Ambion, Thermo Scientific, Waltham, MA, USA). Cells were scraped and transferred to a new tube and tissues were lysed with TissueLyser (Qiagen, Hilden, Germany) before being transferred to a new tube. RNA was then extracted via the Trizol-Chloroform-Isopropanol method, as previously described [24]. RNA concentrations were determined with NanoDrop technology (Thermo Scientific, Waltham, MA, USA) and reverse transcription was performed using a High-Capacity cDNA Reverse Transcription Kit (Applied Biosystems™, Waltham, MA, USA). qPCR was performed using PowerUp™ SYBR™ Green (Applied Biosystems™, Waltham, MA, USA) according to the user’s manual (triplicate for each sample). qPCRs were run on QuantStudio™ 5 Real-Time PCR System (Applied Biosystems™, Waltham, MA, USA, 384-well). Primer sequences used for qPCR are indicated in Table S1.

### 2.5. Bioinformatic Analysis

#### 2.5.1. miR-22 Expression in GEO Datasets

The expression of miR-22-3p or *miR22HG* was assessed in Gene Expression Omnibus (GEO) transcriptomic datasets of HCC patient cohorts (non-tumoral hepatic samples vs. HCC samples) or mouse models of hepatic tumor development (non-tumoral hepatic samples vs. HCC samples). Datasets GSE10694, GSE21362, GSE60502, and GSE64041 contained paired non-tumoral and HCC samples. The data of miR-22-3p/*miR22HG* expression profiles were analyzed via the GEO2R online software (https://www.ncbi.nlm.nih.gov/gds/, accessed on 15 April 2022). All information related to the analyzed GEO datasets is indicated in Table S2.

#### 2.5.2. Survival Analysis

The Gepia cancer database (http://gepia.cancer-pku.cn/, accessed on 6 April 2022) was used to analyze the overall and disease-free survival of HCC patients from the TCGA cohort in regards to the level of expression of miR-22 (parameters used: Cutoff—median; Hazards ratio—yes; Confidence interval—95%; Dataset—LIHC). The same database was used to determine the expression of the *miR22HG* gene in 160 non-tumoral liver samples and 369 HCC samples (parameters used: Dataset—LIHC; Log_2_FC cutoff—1; *p*-value cutoff—0.01; Log scale—yes; jitter size—0.4; Matched normal data—match TCGA normal and GTEx data). The database was also used to segregate the HCC samples of the TCGA cohort regarding the stage of the cancer sample and the expression of miR-22 was assessed in each stage group (parameters used: Dataset—LIHC; Major stage—yes; Log Scale—no). Graphical representation and statistical analyses for these data were performed by the Gepia cancer database.

#### 2.5.3. HCC Segregation by Main Mutations

The cBioPortal database (https://www.cbioportal.org/, accessed 8 April 2022) was used to download the mRNA expression and mutation data from the TCGA-LIHC Pan Cancer cohort of patients. HCC samples from patients of the TCGA cohort were segregated based on the main mutations present in the sample (*TP53* and *CTNNB1*) and the expression of miR-22 was assessed in each group. Patients with double mutations were excluded from the analysis. Data was represented using the R software (v4.1.0, creators Ross Ihaka and Robert Gentleman, open-source free software) with packages ggpubr (v0.4.0) and ggplot2 (v3.3.5).

#### 2.5.4. Biological Process Enrichment Analysis

All proteins found significantly upregulated in miR-22KO livers in the proteomic analysis that we previously published (n = 148, miR-22KO versus WT group, FDR ≤ 0.05) [24] were submitted to Gene Ontology (GO) enrichment analysis by biological process (BP). The 10 most significantly enriched processes were represented as a chord plot. This analysis was undertaken using the R software (v4.1.0, creators Ross Ihaka and Robert Gentleman, open-source free software) with packages GOplot v1.0.2, tidyr v1.2.0, dplyr v1.0.8, GO.db v3.13.0, clusterProfiler v4.0.5, and org.Hs.eg.db v3.13.0. The same protocol was used to perform enrichment analysis using a list of proteins significantly downregulated in miR-22KO livers in the proteomic analysis that we previously published (*n* = 183, miR-22KO versus WT group, FDR ≤ 0.05) [24].

#### 2.5.5. HCC-Associated Target Genes of miR-22

The gene names of 148 proteins that were found significantly upregulated in the livers of miR-22KO mice compared to WT mice at 12 weeks of high-fat diet [24] were transcribed from mouse gene name to a human gene name using the Database for Annotation, Visualization, and Integrated Discovery (DAVID) (https://david.ncifcrf.gov/, accessed on 7 January 2022). The list of human genes obtained through DAVID contained 127 candidates (21 genes from the mouse list did not have a specific ortholog in humans or were eliminated as duplicates). These 127 genes were further cross-referenced with a list of genes associated with HCC, obtained from the MetaCore^™^ database (accessed 7 January 2022), and thus we obtained 22 candidates of potential miR-22 targets associated with HCC. Cross-referencing and the Venn diagram of the cross-section were done via the R software (v4.1.0) and the package VennDiagram v1.7.3. These 22 candidates were screened to check whether they are a predicted/validated human/mouse target of miR-22 using the miRWalk database (http://mirwalk.umm.uni-heidelberg.de/, accessed on 14 December 2021). Genes were considered as potential miR-22 targets when they were identified by at least 3 algorithms from the database (algorithms used: miRWalk, Microt4, miRanda, mirbridge, miRDB, miRMap, miRNAMap, Pictar2, PITA, RNA22, RNAhybrid, and Targetscan). Ten of 22 candidates were found to be predicted/validated targets of miR-22, among which 4 were considered as metabolic targets and 6 were considered as non-metabolic targets based on the available literature.

### 2.6. Statistical Analysis

When comparing the statistical significance between 2 groups, normal distribution was assessed (Shapiro) and unpaired student *t*-test was applied. For statistical analysis involving more than 2 groups, one-way or two-way ANOVA, followed by Šídák’s or Tukey’s multiple comparison test, was applied. Tests were conducted using the GraphPad Prism 9 software. *p*-values are represented as follows: * *p* ≤ 0.05; ** *p* ≤ 0.01; *** *p* ≤ 0.001; **** *p* ≤ 0.0001.

## 3. Results

### 3.1. miR-22 Expression Is Downregulated in Hepatocellular Carcinoma (HCC)

**Figure 1:**
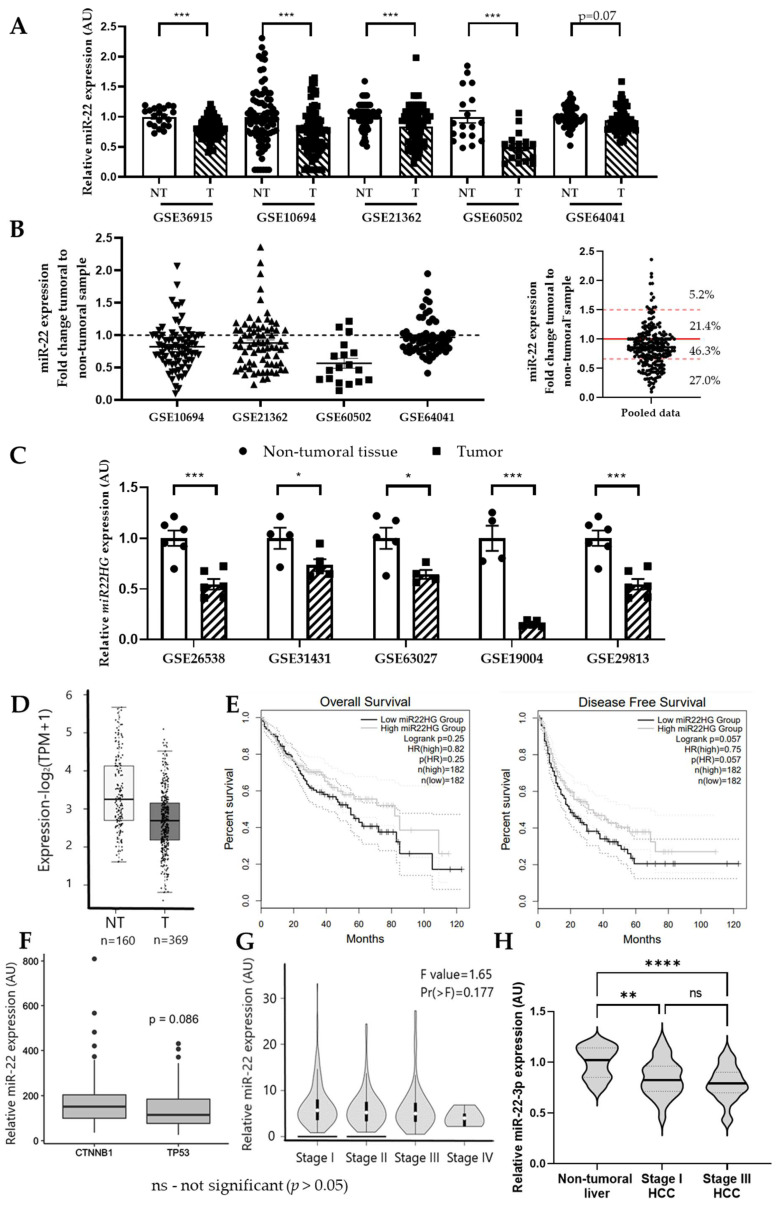
In silico analysis of miR-22 expression in human and rodent hepatocellular carcinoma. (**A**) Analysis of the expression of miR-22-3p or *miR22HG* in Gene Expression Omnibus (GEO) datasets of human HCC patients. Data are represented as fold change between the mean expression in non-tumoral liver (NT, white bars) and tumor (T, bars with stripes) samples (mean +/− SEM). GSE10694 (NT *n* = 78, T *n* = 78), GSE21362 (NT *n* = 73, T *n* = 73), GSE60502 (NT *n* = 18; T *n* = 18), and GSE64041 (NT *n* = 60; T *n* = 60) are human datasets with paired non-tumoral and HCC samples, whereas GSE36915 (NT *n* = 21, T *n* = 68) is a dataset with unpaired non-tumoral and HCC samples. (**B**) Analysis of the individual patient fold change of *miR22HG*/miR-22-3p expression (HCC/non-tumoral sample expression ratio) in paired datasets presented in (**A**) (**left**) and pooled analysis of the fold changes of the 4 GEO datasets (**right**). (**C**) Analysis of the expression of *miR22HG* in Gene Expression Omnibus (GEO) datasets of mouse hepatic cancer models. Data are represented as fold change between the mean expression in non-tumoral liver (NT, white bars) and tumor (T, bars with stripes) samples (mean +/− SEM). Mouse datasets used for the analysis: GSE26538 (NT *n* = 6 T *n* = 6); GSE31431 (NT *n* = 4, T *n* = 5); GSE63027 (NT *n* = 5, T *n* = 4); GSE19004 (NT *n* = 4, T *n* = 5); GSE29813 (NT *n* = 6, T *n* = 6). (**D**) Expression of *miR22HG* in non-tumoral liver samples (light gray bar) and HCC samples (dark gray bar) of the TCGA-LIHC cohort of patients (NT *n* = 160, T *n* = 369). (**E**) Kaplan Meier representation of the overall survival and disease-free survival rates of patients with low *miR22HG* expression in HCC biopsy (black line, *n* = 182) vs. patients with high expression of *miR22HG* (gray line, *n* = 182). (**F**) Expression of *miR22HG* in HCC samples of the TCGA-LIHC cohort of patients presenting either β–catenin (*CTNNB1*) or p53 (*TP53*) mutation. (**G**) Expression of *miR22HG* in HCC samples of the TCGA-LIHC cohort of patients segregated with regards to the stage of the HCC (Gepia database, accessed 6 April 2022). (**H**) Expression of miR-22-3p in HCC samples of the GEO dataset GSE36915 of HCC patients segregated with regards to the stage of the HCC (NT *n* = 21, T-Stage-I *n* = 26; T-Stage-III *n* = 42). For (**A**,**C**,**D**,**F**) Student’s *t*-test was performed. For (**D**,**E**,**G**), statistical analyses were performed by the Gepia cancer database software. For (**H**), one-way ANOVA was performed. *p*-values are represented as follows: * *p* ≤ 0.05; ** *p* ≤ 0.01; *** *p* ≤ 0.001; **** *p* ≤ 0.0001. *ns*—not-significant (*p* > 0.05).

MiR-22 expression was previously reported to be decreased in HCC biopsies from different patient cohorts [28,32,33]. In silico analysis of publicly available GEO datasets of human non-tumoral hepatic samples and HCC further indicated miR-22 downregulation in HCC from five other different human patient cohorts (Figure 1A, Table S2). Of note, the first two datasets reported in Figure 1A (GSE36915 and GSE10694) represent the expression of the miR-22 host gene (*miR22HG*) rather than the mature hsa-miR-22-3p. Nevertheless, the expressions of *miR22HG* and hsa-miR-22-3p were previously shown to tightly correlate in HCC [38,39]. All datasets, with the exception of GSE36915, contained paired non-tumoral and HCC samples of patients. The expression of *miR22HG*/miR-22-3p in these four datasets (GSE10694, GSE21362, GSE60502, GSE64041) was further reported as a fold change between the HCC sample and the non-tumoral hepatic tissue of each individual patient (Figure 1B, left panel). Pooled analysis of the four datasets revealed that nearly 70% of the patients presented a fold change ≤1 and 27% of all patients had a fold change ≤0.66, illustrating the high proportion of patients with low miR-22 expression (Figure 1B, right panel). Significantly decreased expression of *miR22HG* was further observed in five different GEO datasets of different mouse models of HCC (Figure 1C, Table S2).

Corroborating these observations, HCC from patients included in the cancer genome atlas (TCGA cohort, liver hepatocellular carcinoma—LIHC group) [40] also display a significant downregulation of the miR-22 host gene *miR22HG* as compared to non-tumoral liver biopsies (Figure 1D). The same patients were further segregated into two groups (low *miR22HG* vs. high *miR22HG* expression in HCC sample) and their survival rates were assessed. Overall survival showed a decreasing trend in patients with lower *miR22HG* expression in tumors, whereas the disease-free survival was significantly worse in this group, compared to that of patients with HCC expressing higher levels of *miR22HG* (Figure 1E). Similarly, survival data from the oncomiR database (oncomir.org) revealed that living HCC patients present significantly higher levels of miR-22-3p expression (living Log2 mean expression = 17.20) compared to deceased patients (deceased Log2 mean expression = 16.99; *t*-test *p*-value = 3.96 × 10^−3^; *t*-test FDR = 7.93 × 10^−2^).

The prognosis of patients with HCC is partly dependent on the type of mutations present in HCC, with patients bearing β-catenin (*CTNNB1*) mutations having a better prognosis than those bearing p53 (*TP53*) mutations [41]. Confirming our survival analyses, downregulation of miR-22-3p/*miR22HG* was mostly observed in HCC-bearing *TP53* mutations (*p* = 0.08) but not *CTNNB1* mutations (Figure 1F and Figure S1). Finally, we found that miR-22 expression was unrelated to the tumor staging (classifying HCC based on the size of the tumor, tumor nodule number, and tumor invasion state) in either the TCGA-LIHC patient cohort (Figure 1G), or in another patient cohort (GSE36915 dataset) segregating HCC biopsies in stage I versus stage III (Figure 1H), suggesting that miR-22 downregulation could be an early event in hepatocarcinogenesis.

### 3.2. miR-22 Deficiency Fosters Tumor Development In Vivo

In order to assess the functional in vivo role of miR-22 in HCC initiation and development, hepatic carcinogenesis was induced by injection of diethylnitrosamine (DEN) in 15-day-old miR-22KO and wild-type (WT) littermate mice. At adulthood (two months of age), miR22KO and WT mice were randomized into two different groups fed either a standard chow diet (CD group) or an obesogenic high fat-enriched diet (HFD group) for 9 months until the age of 11 months before sacrifice and ex vivo analyses of explanted tissues.

**Figure 2:**
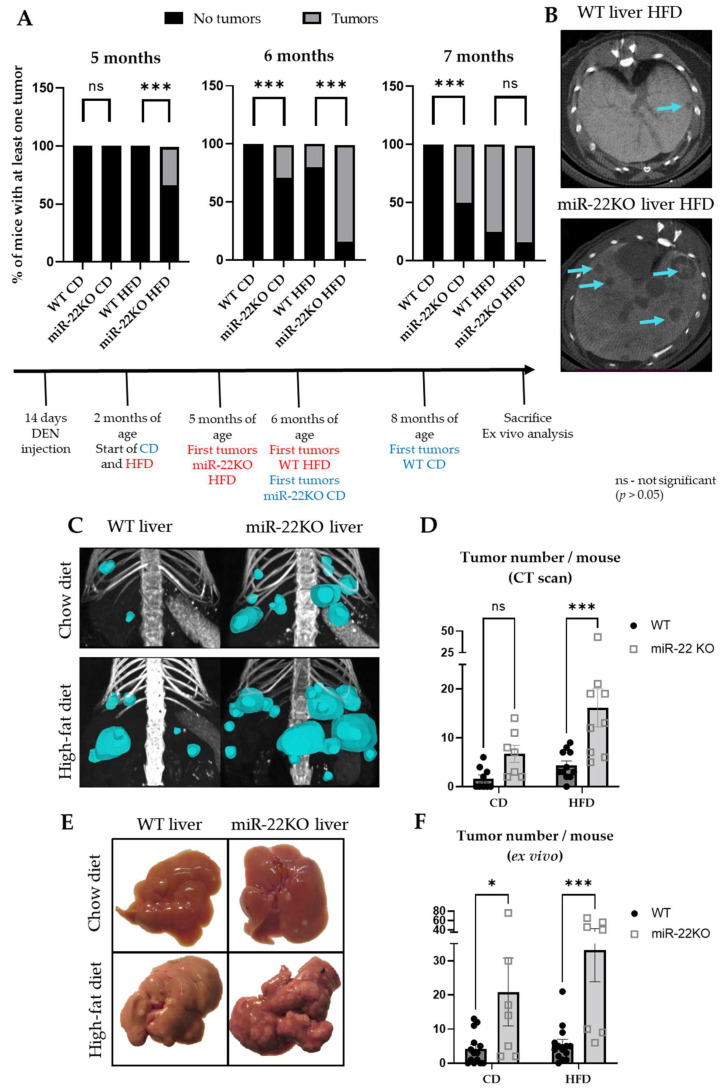
Initiation and progression of hepatocarcinogenesis in miR-22KO and WT mice. (**A**) CT-scan assessment of hepatic tumor incidence over time; graphs represent the percentage of WT and miR-22KO mice under chow diet (CD) or high-fat diet (HFD) bearing at least one tumor at 5, 6, or 7 months of age (*n* = 3–6 mice/group). (**B**) Representative images of liver/tumor sections of WT and miR-22KO mice at 6 months of age (axial view, CT-scan imaging). Tumors are indicated with blue arrows. (**C**) 3D representative images of the tumors (blue) and (**D**) number of tumors per mouse detected during CT-scan analysis in WT and miR-22KO mice under CD or HFD at 11 months of age (*n* = 7–11 mice/group). (**E**) Representative images of the livers and (**F**) number of tumors per mouse detected during ex vivo analysis of miR-22KO mice and WT littermates under standard chow diet (CD) or high-fat diet (HFD), at time of sacrifice (*n* = 7–14 mice/group). Of note, 2 mice from the miR-22KO-HFD group were excluded from panel F, as the number of tumors observed ex vivo was too high and individual tumors were not discernable, thus not allowing a precise counting of tumors. For panel (**A**), Fisher’s tests were performed to analyze statistical significance. For (**D**,**F**), data are represented as mean +/− SEM and two-way ANOVA (multiple comparisons) were performed. *p*-values are represented as follows: * *p* ≤ 0.05; *** *p* ≤ 0.001.

Tumor incidence and progression were assessed monthly by CT-scan analysis in a subset of mice from the four groups starting from the age of 5 months (after 3 months of differential diets). In mice fed a normal chow diet, the first tumors were detected at the age of 8 months in WT mice versus 6 months of age in miR22KO mice. The obesogenic diet (HFD) further accelerated tumor initiation in both groups, with the first tumors appearing at the age of 6 months in WT mice versus 5 months in miR22KO mice (Figure 2A,B). CT scan analyses just before sacrifice at 11 months of age (9 months of differential diets) confirmed, as previously described [42], the tumor-promoting effect of an obesogenic high fat-containing diet in the liver (WT mice fed a CD versus HFD, *p* = 0.03; Figure 2C,D). Finally, miR-22KO mice fed an HFD developed significantly more tumors than WT littermates fed the same diet (Figure 2C,D). MiR-22 deficiency also tends to foster carcinogenesis in mice fed a CD. (Figure 2C,D). Ex vivo analysis of apparent tumoral nodules at the surface of explanted livers further confirmed the CT-scan analyses at sacrifice by showing an increased number of nodules present on the liver of miR-22KO mice, as compared to WT littermates, regardless of the type of diet (Figure 2E,F). Of note, the obesogenic diet to which mice were submitted does not induce tumorigenesis in the time frame of our experimental set up [17,43,44] and miR22KO mice do not develop hepatic tumors spontaneously with ageing, as monitored until at least 18 months of age.

Together these data clearly demonstrated that miR-22 deficiency in vivo fosters tumor initiation in the liver of mice, a pathological process further exacerbated in mice developing NAFLD and obesity.

### 3.3. miR-22 Loss Promotes Histopathological Features of NASH and the Development of More Undifferentiated Tumoral Nodules

**Figure 3:**
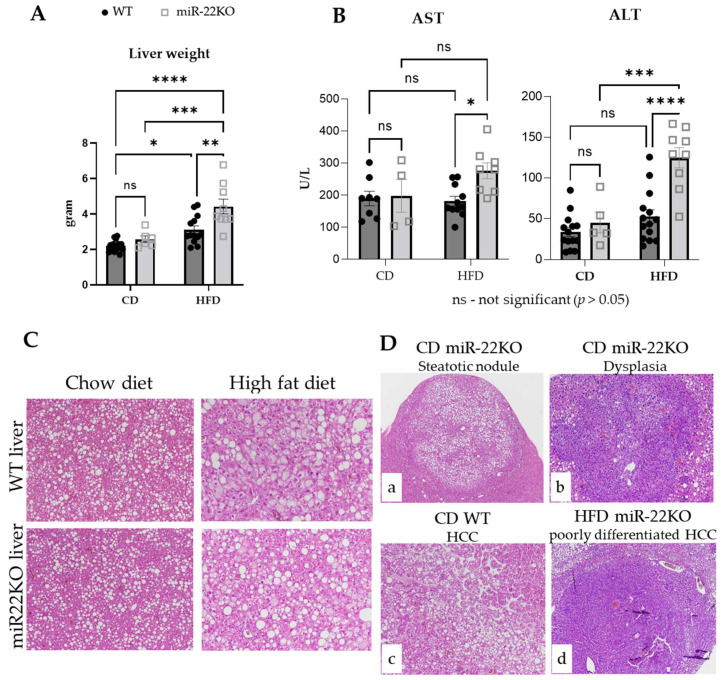
Ex vivo and histological analysis of livers and tumors of miR-22KO and WT mice. (**A**) Liver weight (*n* = 6–14/group), (**B**) plasmatic aspartate aminotransferase (AST, *n* = 4–11/group) and alanine aminotransferase (ALT, *n* = 5–14/group) levels of miR-22KO mice and WT littermates under standard chow diet (CD) or high-fat diet (HFD), at time of sacrifice. Data are represented as mean +/− SEM. Two-way ANOVA (multiple comparisons) were performed. *p*-values are represented as follows: * *p* ≤ 0.05; ** *p* ≤ 0.01; *** *p* ≤ 0.001; **** *p* ≤ 0.0001. (**C**) Representative H&E-stained histological images of non-tumoral livers of WT and miR-22KO mice under CD (magnification ×10) and HFD (magnification ×20). (**D**) Representative histological images of tumors with H&E staining ((**a**)—Steatotic nodule in a miR-22KO mouse under CD, magnification ×4; (**b**)—Dysplasia in a miR-22KO mouse under CD, magnification ×10; (**c**)—HCC in a WT mouse under CD, magnification ×10; (**d**)—poorly differentiated HCC in a miR-22KO mouse under HFD, magnification ×4).

At sacrifice, liver weight was unchanged in miR-22KO mice fed CD, as compared to WT littermates. However, under HFD, hepatomegaly was exacerbated in miR-22KO mice compared to WT mice (Figure 3A). Furthermore, higher blood levels of transaminase enzymes (both AST and ALT), which are usually not associated with HCC development, were observed with miR-22 deficiency in obese mice and the long-term effects of DEN exposure (Figure 3B), reflecting hereby important hepatic stress/injury through mechanisms potentially distinct from cell death [45,46,47,48,49,50,51]. Other metabolic plasma markers were normal except cholesterol levels, which were abnormally elevated in the plasma of miR22KO mice fed an HFD, suggesting major metabolic alterations of the lipid metabolism in these conditions (Figure S2).

Histopathological analyses of tumoral and non-tumoral hepatic tissues were performed by an expert liver pathologist and indicated that steatosis and hepatocyte ballooning were similar in 11-month-old WT and miR22KO mice fed a CD. However, 11-month-old mice fed an HFD displayed less steatosis, but much more hepatocyte ballooning than CD-fed mice, revealing a more severe NASH phenotype under this obesogenic diet (Figure 3C). Steatotic nodules were observed in both WT and miR22KO mice regardless of the regimen, but basophilic nodules with small hepatocytes suggesting dysplasia were also observed in miR22KO mice under CD (Figure 3D). Finally, HCCs were found in all mouse groups. However, the frequency of HCC nodules was much higher in miR22KO mice under HFD, which also often exhibited poorly differentiated features (Figure 3D). Additional analyses of albumin and α-antitrypsin expression in paired non-tumoral liver tissues, and respective tumors in each group, further confirmed that HCCs in miR22KO mice fed an HFD were poorly differentiated, as compared to those developing in WT mice under the same regimen. (Figure S3A). A similar trend was also observed in miR-22KO tumors under CD. Finally, glypican 3 positive nodules (further discriminating between HCC and dysplastic nodules) were more frequently observed in mice fed an HFD, but with no differences in regards to the presence or absence of miR-22 (Figure S3B). Of note, mixed hepatobiliary, or biliary, tumors were not observed in any of the groups or diets. Altogether, these data indicate that miR-22 deficiency not only exacerbates specific histopathological features associated with diet-induced metabolic disorders in the liver, but it also fosters dedifferentiation and severity of DEN-induced HCC in mouse livers.

### 3.4. miR-22 Regulates Hepatic Metabolism through Multiple Mechanisms

**Figure 4:**
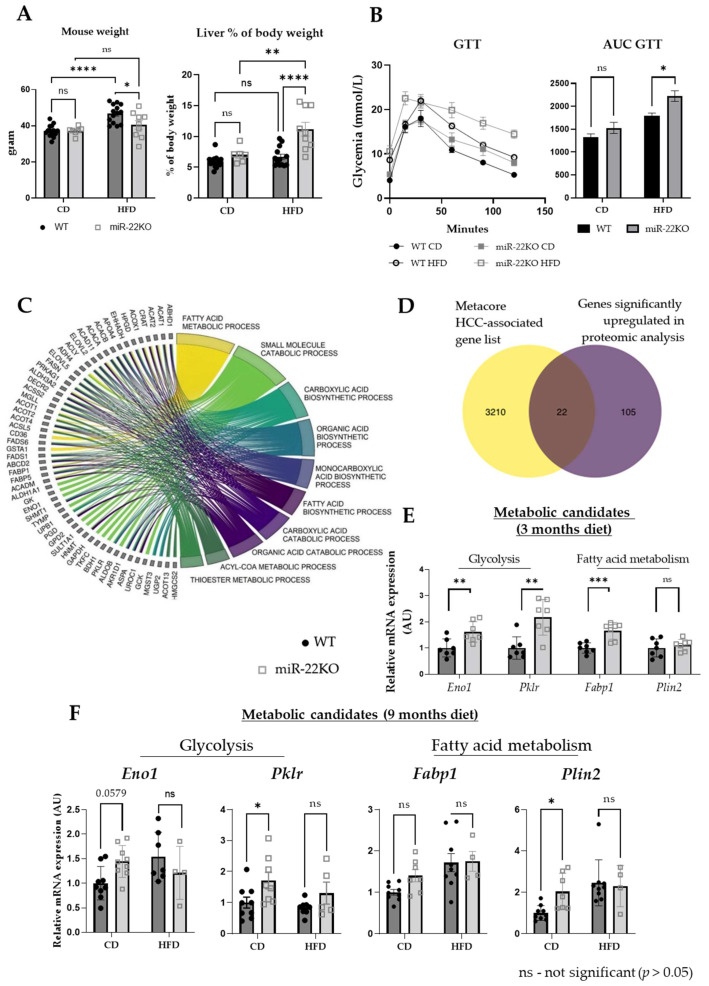
Expression of potential metabolism-related target genes of miR-22. (**A**) Body weight (*n* = 7–14/group) and liver to body weight ratio (*n* = 6–14/group) of miR-22KO mice and WT littermates under standard chow diet (CD) or high-fat diet (HFD), at time of sacrifice. (**B**) Glucose tolerance test (GTT) and associated area under the curve (AUC) in miR-22KO mice and WT littermates under standard chow diet (CD) or high-fat diet (HFD), at 5 months (*n* = 3–5/group). (**C**) Chord plot representation of Gene Ontology (GO) enrichment by biological process analysis performed on significantly upregulated proteins found in the proteomics of livers of miR-22KO vs. WT mice challenged with HFD for 3 months [24]. (**D**) Cross-referencing of a list of genes associated with HCC (list generated by MetaCore database), with a list of proteins that were upregulated in the proteomics of livers of miR-22KO vs. WT mice challenged with HFD for 3 months [24]. RT-qPCR analysis of mRNA expression of *Eno1*, *Pklr*, *Fabp1*, and *Plin2* in (**E**) the livers of miR-22KO and WT mice under HFD for 3 months (diet began at 2 months of age, *n* = 7/group) and in (**F**) the livers of DEN-treated miR-22KO and WT mice under CD or HFD (diet starting at 2 months of age and lasted 9 months, *n* = 4–9 mice/group). Data in (**E**,**F**) are represented as fold change of the mean expression in each group +/− SEM. Unpaired Student *t*-tests were applied for (**E**) and two-way ANOVA (multiple comparison) was applied for (**A**,**B**,**F**). *p*-values are represented as follows: * *p* ≤ 0.05; ** *p* ≤ 0.01; *** *p* ≤ 0.001; **** *p* ≤ 0.0001.

The impact of miR-22 deficiency on whole-body homeostasis appears complex and multifactorial. We previously showed that miR22KO mice fed an HFD for 3 months were more obese and insulin resistant than WT mice under the same regimen [24]. However, over the 9 months of long-term HFD diet following HCC induction by DEN, the obesogenic effect of the HFD was lost in miR22KO mice (Figure 4A). Although this phenotypic trait was attenuated with ageing, miR22KO mice still display a higher liver-to-body weight ratio and a decreased glucose tolerance, as compared to WT mice (Figure 3A and Figure 4A,B). The early onset of tumor appearance in miR22KO mice fed an HFD diet suggested that miR-22 deficiency upon metabolic stress primes hepatocytes for carcinogenesis. In order to more precisely pinpoint miR-22-dependent cellular processes fostering hepatocarcinogenesis, we took advantage of our previous proteomic analysis of hepatic tissues explanted from WT and miR22KO mice fed an HFD for 3 months before sacrifice at 5 months of age [24]. Gene Ontology (GO) enrichment analysis by biological processes (Figure 4C, Table S3) showed that proteins significantly upregulated in the livers of miR-22KO mice were mostly involved in hepatic metabolic functions (e.g., fatty acid metabolism, carboxylic acid biosynthesis, and catabolism), a result that was further confirmed by String enrichment analyses (Table S4). Of note, consistent with the increased blood cholesterol found in miR-22KO mice under HFD (Figure S2), but likely not a direct consequence of miR-22 deficiency, GO enrichment analysis suggested that, on the contrary, downregulated proteins in miR-22KO livers point to sterol- and cholesterol-related metabolic processes (Table S5).

Proteins upregulated in miR-22KO hepatic tissues, and thus potentially directly targeted by miR-22, were then cross-referenced with HCC-associated genes (MetaCore database). A total of 22 proteins were recognized as cancer-related factors upregulated in hepatic tissues of miR22KO mice fed an HFD (Figure 4D, Table S6), among which 10 were further identified as potential/validated miR-22 direct target genes in human or mouse models by the miRWalk database (red name in Table S6). Four of these 10 genes (outlined in yellow in Table S6), i.e., enolase 1 (*Eno1)*, pyruvate kinase L/R (*Pklr*), fatty acid binding protein 1 (*Fabp1*), and perilipin 2 (*Plin2*), are key relevant actors in the hepatic glucose/lipid metabolism and are also reported to promote HCC development [52,53,54,55]. The six other identified factors (outlined in green in Table S6), i.e., thrombospondin 1 *(Tsp1*), galectin 1 (*Lgals1*), glutathione S-transferase mu 3 (*Gstm3*), glutamate-cysteine ligase catalytic subunit (*Gclc*), polymeric immunoglobulin receptor (*Pigr*), and 15-hydroxyprostaglandin dehydrogenase (*Hpgd*) are not key factors governing hepatic metabolism, but some of them were previously identified as potent oncogenes (e.g., *Lgals1* [56], *Pigr* [57]).

Regarding miR-22 targets in metabolism, *Eno1* and *Pklr* regulate glycolysis [58,59], while *Fabp1* and *Plin2* are key actors of fatty acid trafficking and storage in hepatocytes [60,61]. We confirmed that these four candidates were also upregulated at the mRNA levels, with the exception of *Plin2*, in hepatic tissues processed for our proteomic analysis (5-month-old miR22KO mice fed for 3 months an HFD, Figure 4E), thus suggesting that miR-22 may downregulate most of these factors by inducing their mRNAs’ degradation. Although expression of these genes was also increased in 11-month-old DEN-treated miR22KO mice fed a CD, upregulation of these factors was no longer observed at the mRNA level in 11-month-old DEN-treated miR22KO mice fed an HFD (Figure 4F), suggesting that the increased liver carcinogenesis observed in these mice also affects metabolism with aging, in addition to body weight (Figure 4A).

**Figure 5:**
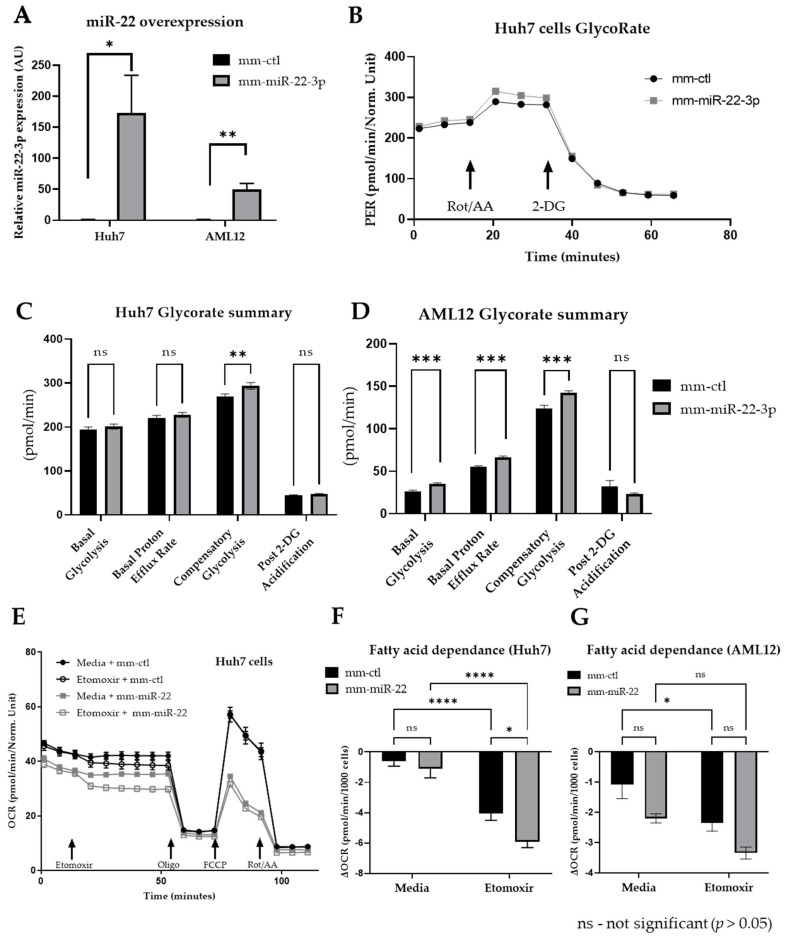
Glycolysis rate and fatty acid-dependence of miR-22-overexpressing hepatic cells. (**A**) RT-qPCR analysis of miR-22-3p expression in Huh7 and AML12 cells transfected with 20 nM of miR-22-3p-mimicking oligonucleotides (mm-miR-22-3p) or control oligonucleotides (mm-ctl), 48 h post-transfection. (**B**) Representative traces of glycolysis rate measurement (GlycoRate, Seahorse Agilent technology) in Huh7 cells transfected or not with miR-22-3p-mimicking oligonucleotides for 48 h. GlycoRate quantification in (**C**) Huh7 and (**D**) AML12 cells transfected with 20 nM of mm-miR-22-3p or mm-ctl, 48 h post-transfection. (**E**) Representative traces of long-chain fatty acid oxidation level determined by measuring the oxygen consumption rate (OCR) measurement (Seahorse Agilent technology) in the presence/absence of Etomoxir (inhibitor of fatty acid transport to mitochondria) in Huh7 cells overexpressing miR-22-3p or not, 48 h post-transfection. Fatty acid dependence (acute response to Etomoxir) in (**F**) Huh7 and (**G**) AML12 cells from the previous panel. Data are represented as mean +/− SEM. Statistical relevance of (**A**,**C**,**D**) was analyzed with Student’s *t*-tests and two-way ANOVA (multiple comparisons) was used in (**F**,**G**). *p*-values are represented as follows: * *p* ≤ 0.05; ** *p* ≤ 0.01; *** *p* ≤ 0.001; **** *p* ≤ 0.0001.

The functional role of miR-22 in glucose and lipid metabolism was further investigated in hepatic cell lines. Human Huh7 hepatoma cells and mouse AML12 immortalized hepatic cells, widely used for metabolic analyses and both expressing low levels of miR-22 as compared to primary hepatocytes [24], were transfected with synthetic oligonucleotides mimicking miR-22-3p (mm-miR-22-3p, Figure 5A), and then glucose and lipid consumption were assessed by Seahorse analyses (Seahorse Agilent technology). Glycolysis rate measurement (GlycoRate test) showed that miR-22-3p promotes compensatory glycolysis in Huh7 cells (Figure 5B,C). The same effect of miR-22 mimics was observed in AML12, which display in addition a miR-22-dependent increased basal glycolysis and basal proton efflux rate (Figure 5D). These data are consistent with our previously published GlycoStress tests in miR-22-3p overexpressing Huh7 cells, which indicate an upregulated glycolysis rate and capacity induced by miR-22 expression [24].

The contribution of fatty acids to mitochondrial respiration was further assessed in Huh7 and AML12 cells transfected with miR-22-3p, by performing a Seahorse-based long-chain fatty acid oxidation stress test following a blockage of fatty acid transport to the mitochondria by etomoxir (a blocker of carnitine palmitoyltransferase I-CPT1). We found that miR-22 expression decreases mitochondrial respiration in Huh7 cells by restraining the maximal respiration capacity, mitochondrial ATP production, and spare respiration capacity (Figure 5E, Figure S4). Importantly, the acute response to etomoxir, indicated that miR-22 in Huh7 cells induces a fatty acids dependence of the mitochondrial respiration (Figure 5F). Similar data, although less clear-cut, were observed in AML12 cells expressing miR-22-3p (Figure 5G).

Together, these data indicate that miR-22 regulates key factors of the lipid and glucose metabolism which functionally impacts mitochondrial activity and, therefore, the bioenergetics of cancer cells.

### 3.5. Loss of miR-22 Increases the Expression of Key Target Oncogenes

**Figure 6:**
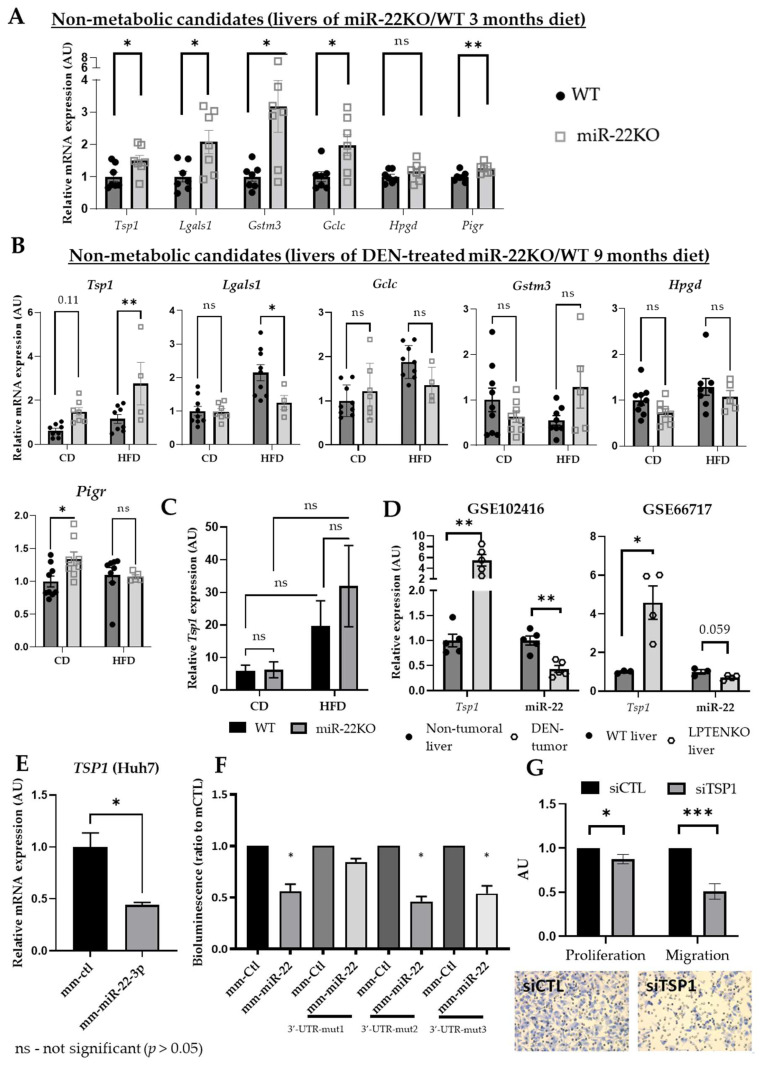
MiR-22 regulates cancer hallmarks through non-metabolic target genes. RT-qPCR analysis of mRNA expression of *Tsp1*, *Lgals1*, *Gstm3*, *Gclc*, *Hpgd*, and *Pigr* in (**A**) the livers of miR-22KO and WT mice under HFD for 3 months (diet began at 2 months of age, *n* = 7 mice/group) and (**B**) the livers of DEN-treated miR-22KO and WT mice under CD or HFD for 9 months (diet began at 2 months of age, *n* = 4–9 mice/group). (**C**) *Tsp1* mRNA expression in the tumor samples obtained from DEN-treated miR-22KO and WT mice under CD or HFD for 9 months (*n* = 4–10 tumors/group). (**D**) Relative *Tsp1* and miR-22 expression levels in GEO datasets GSE102416 (*n* = 5/group) and GSE 66,717 (*n* = 3–4/group). (**E**) RT-qPCR analysis of mRNA expression of *TSP1* in Huh7 cells transfected with 20 nM of miR-22-3p-mimicking oligonucleotides (mm-miR-22-3p) or control oligonucleotides (mm-ctl), 48 h post-transfection (*n* = 3). (**F**) Inhibition of the luciferase activity of pLightSwitch-3UTR-TSP1, bearing or not mutations (Mut1, Mut2, or Mut3) in each predicted seed sequence for miR-22, in HepG2 cells treated with miR-22 mimics for 48 h. (**G**) Proliferation rate and migration capacity (Boyden chamber assay) of HepG2 cells transfected with either control siRNA or siRNA targeting TSP1 expression (siTSP1) at 48 h post-transfection. Data are represented as fold change, mean +/− SEM. Unpaired Student *t*-tests were applied in (**A**) and (**D**–**G**). Two-way ANOVA (multiple comparison test) were conducted for (**B**,**C**). *p*-values are represented as follows: * *p* ≤ 0.05; ** *p* ≤ 0.01; *** *p* ≤ 0.001.

In addition to driving metabolic switches promoting cancer, our proteomic data indicate that miR-22 deficiency may also promote carcinogenesis by not sufficiently restraining the expression and activity of relevant oncogenes. In this regard, galectin-1, identified by our proteomic analysis (Figure 4, Table S6), was previously reported to be a direct target of miR-22 in vitro [31] and to represent an important oncogene promoting liver cancer [56]. We therefore first assessed whether the six potential miR-22 target genes classified as non-metabolic targets (*Tsp1*, *Lgals1*, *Gstm3*, *Gclc*, *Pigr*, and *Hpgd*, Table S6, green) were also upregulated at the mRNA level in the liver of miR22KO mice. All genes, except *Hpgd*, were significantly upregulated in non-tumoral hepatic tissues of 5-month-old miR22KO mice fed an HFD for 3 months (Figure 6A); however, in non-tumoral liver tissues of 11-month-old DEN-treated miR22KO mice fed an HFD for 9 months, only *Tsp1* mRNA expression was found to still be upregulated (Figure 6B). Of note, metabolic stress induced by the HFD feeding per se promoted *Tsp1* upregulation in tumors and appears to be required to further allow miR-22-dependent *Tsp1* upregulation (Figure 6C), although miR-22 expression was significantly downregulated in tumors from DEN-treated WT mice fed either a CD or HFD diet (Figure S5).

In silico analyses of publicly available mouse datasets further support *Tsp1* as a direct target of miR-22 under stress conditions, since mRNA expression of *Tsp1* negatively correlates with miR-22 expression. This was observed in DEN-induced liver tumors as compared to non-tumoral tissues (GSE102416), and in liver-specific PTEN knockout mice, a mouse model of spontaneous NAFLD/NASH development triggering HCC with ageing (GSE66717) (Figure 6D). We further observed that, consistent with their low miR-22 expression [24], mouse and human hepatic cancer cells display a significant *TSP1*/Tsp1 upregulation, as compared to primary hepatocytes (Figure S6A,B). *TSP1* expression was also significantly downregulated in Huh7 hepatic cells expressing synthetic miR-22-mimicking nucleotides (Figure 6E). The direct and specific targeting of TSP1’s 3′-UTR sequence by miR-22 was demonstrated through luciferase-based 3′-UTR gene reporter analysis in HepG2 cells treated with miR-22-mimicking nucleotides (Figure 6F). Moreover, mutations of the three different predicted canonical and non-canonical seed sequences (binding sites for miR-22) in the TSP1 3′-UTR of the pLightSwitch-3UTR-TSP1 vector further validated the functional relevance of only the first seed sequence in the 3′-UTR of TSP1 (WT site 1), over the three miR-22 seed sequences predicted by the miRWalk2.0 database, as a direct binding site for miR-22 in hepatoma cells (Figure 6F and Figure S7).

Finally, although *Lgals1* was previously identified as a direct target of miR-22 in other cells [31], overexpression of miR-22 in Huh7 cells did not downregulate *LGALS1* mRNA expression, nor those of *GSTM3*, *GCLC*, or *PIGR* (Figure S8A).

To gain insights into the role of TSP1 in cancer-related hallmarks, we further investigated proliferation and invasion/migration properties of Huh7 and HepG2 cells. These cells display only a 4–6-fold upregulation of TSP1 (as compared to other cancer cell lines, Figure S6A,B), which allow us to efficiently downregulate it through an siRNA-based strategy, and are very well characterized for their metabolism and cancer-promoting cellular processes. As expected based on our in vivo analyses (Figure 2), overexpression of miR-22 in Huh7 cells led to a significantly decreased proliferation rate (Figure S8B), an effect further confirmed in a second human hepatic cancer cell line, i.e., HepG2 hepatoma cells (Figure S8B). Consistent with an oncogenic role of TSP1, silencing of *TSP1* by specific siRNAs in HepG2 cells led to a decreased proliferation, in addition to decreased capacity for migration (Figure 6G).

Altogether, these data showed that besides a strong effect on the bioenergetic metabolism, miR-22 can also affect tumor development by specifically modulating the expression of potential oncogenic factors such as TSP1.

## 4. Discussion

With NAFLD, hepatic inflammation and metabolic disorders provide a pathological microenvironment prone to the occurrence of genetic mutations associated with HCC development (3,4,6). In this context, the abnormal expression of specific miRNAs plays an important role in metabolic disturbances, inflammation, and carcinogenesis by altering the expression of whole networks of genes regulating these processes [16,62]. miR-22, in particular, is one of the most highly expressed microRNAs in the liver that is strongly downregulated in human HCC (28,32,33). Herein, we investigated the in vivo and in vitro roles of this miR-22 downregulation in the development of HCC. Our analyses strongly support a significant downregulation of miR-22 in human HCC, and in all mouse models of this cancer that we investigated. In vivo analyses of HCC induction in genetically depleted miR-22 mice further point to a tumor-suppressive role of this miRNA, particularly when NAFLD is induced by a long-term fat-enriched dietary regimen. Finally, proteomic analyses of hepatic tissues before tumor development indicated that miR-22 deficiency induces pleiotropic metabolic alterations of the glucose and lipid metabolism, in addition to deregulated expression of cancer-promoting factors, which altogether likely contribute to foster carcinogenesis in a NAFLD context.

MiR-22 expression is highly specific to the tissue or cell, but the molecular mechanisms ensuring this expression specificity are unclear [63,64]. MiR-22 is also differentially expressed in distinct human cancers [65] and downregulated in HCC, but the underlying molecular mechanisms of this decrease remain unknown. Previous studies investigating the regulation of miR-22 expression in different cells/tissues/organs, pathophysiological situations, and/or diseases, have revealed a plethora of different and heterogenous mechanisms regulating miR-22 expression. MiR-22 expression is, for example, affected by various chemical agents such as phorbol-12-myristate-13-acetate [66], 12-o-tetradecanoylphorbol-13-acetate [67], polyinosinic-polycytidylic acid (poly(I:C)) [68], catalpol [69], sodium butyrate [70,71], endosulfan [72], short-chain fatty acids (e.g., propionate and valerate) [73], retinoic acid [73], bile acids [70,74], or exendin-4 [75]. Inflammatory cytokines, e.g., IL-1α [36], or metabolites, such as extracellular ATP/UTP [76], can also impact its expression. The ribosome protein L29 (RPL29) was also reported as an intracellular factor regulating miR-22 levels [64]. Finally, several studies indicated that the expression of miR-22 is regulated transcriptionally, along with its host gene, by numerous transcription factors including NF-κB [77], p53 [78], Fos-B [66], c-Fos [79], PU.1 [80], STAT3 [81], STAT5 [81], Nrf1α, and Nrf2 [82]. MiR-22 expression level is further dependent on the activation of different intracellular signaling pathways such as those signaling through AKT [83], Jak3 [81], or testosterone [84]. Of note, viral infections also affect miR-22 expression, such as infection by influenza viruses, which modulate miR-22 expression likely through SP1- and c-Myc-dependent transcriptional mechanisms [85]. From this extensive literature, it appears that downregulation of miR-22 in hepatic tumors can result from multiple mechanisms, which can also be different from one tumor to the other in the same liver, given the high heterogeneity of HCC nodules.

In this regard, the use of miR-22 genetically deficient mice has a major advantage for assessing its role in vivo without worrying about the multiple mechanisms, which may potentially over-activate remnant miR-22 when it is only downregulated by the administration of antagomiRs or shRNAs. Since the miR22KO mice used in this study are total constitutive knockout mice, we can, however, not exclude the idea that inhibition of miR-22-dependent mechanisms in other organs may, for example through inter-organ communication, contribute to some extent to the increased hepatic carcinogenesis observed in miR22KO mice. However, based on the fact that (i) mouse hepatocytes express very high levels of miR-22, as compared to other cells/tissues (shown in our previous study [24], (ii) DEN mostly induces mutagenesis and carcinogenesis in hepatocytes [86], and (iii) our proteomic analyses of hepatic tissues indicate profound changes in the liver proteome of cancer-related factors, and due to the effects of miR-22 modulation in vitro in hepatic cancer cells (Figure 4, Figure 5 and Figure 6), it is likely that the loss of miR-22 in hepatocytes plays a preponderant role in the increased hepatic carcinogenesis that we observed.

As expected from previous in vitro studies (reviewed in [65]), hepatocarcinogenesis in vivo is exacerbated in the absence of miR-22, a feature that was further promoted with diet-induced NAFLD. We observed, however, that most tumor initiation is accelerated by miR-22 deficiency, as suggested by the higher tumor incidence and an earlier tumor onset in miR22KO mice fed a standard or HFD. Regarding tumor progression and malignancy, analyses of the human cohorts performed in this study did not allow us to conclude that the downregulation of miR-22 expression/activity correlates with the tumoral stage in humans. However, miR-22 downregulation was associated with poor prognosis and survival and with p53 (*TP53*) mutations in human cohorts, while our histopathological analyses of tumors in our mouse model revealed a higher incidence of HCC with poorly differentiated features in miR-22KO mice compared to WT littermates. Based on these data, miR-22 downregulation may also correlate with the aggressiveness of the tumors, but additional analyses are now required to firmly establish miR-22 expression in tumoral tissues, or, more ideally, in body fluids, as a diagnostic/prognostic biomarker of HCC.

Based on miRNA-specific 3′UTR seed sequence prediction algorithms and previous studies, miR-22 has several thousand predicted/validated cellular targets [24]. It is therefore highly probable that the increased hepatic carcinogenesis associated with miR-22 deficiency results from numerous altered cancer-promoting cellular processes that are normally finely tuned by miR-22. An additional complexity to identifying relevant miRNA targets in pathological processes lies in the fact that the miRNA functions are (i) cell/tissue specific, (ii) highly dependent on the relative expression of their targets in the same cell/tissue, (iii) strongly influenced by the metabolic and stress status of the cell/tissue, and (iv) inhibited or promoted by various cell/tissue-specific factors (e.g., long non-coding and circular RNAs, pseudogenes) [16,87,88,89,90]. Finally, a major feature of microRNAs is that they regulate gene expression post-transcriptionally by either degrading mRNAs or by blocking their translation. Therefore, proteomic mass spectrometry analyses are more relevant to identify potential targets of a specific miRNA than transcriptomic analyses, which do not reflect mRNA translational blockades [89]. In contrast, the disadvantage of proteomic mass spectrometry analyses resides in their low sensitivity, which restrains protein identification and quantifications to a couple of thousand cellular factors [91]. In spite of this limitation, we have used this approach to find relevant targets of miR-22 and pathways deregulated by miR-22 deficiency early before tumor onset, but which may be of importance for the observed increased carcinogenesis. Based on these analyses, we identified significant changes in important metabolic factors regulating key metabolic processes such as fatty acid metabolism (e.g., perilipin 2 (*Plin2*), fatty acid binding protein 1 (*Fabp1*)) and glycolysis (e.g., pyruvate kinase L/R (*Pklr*), enolase 1 (*Eno1*)), and other potential miR-22 targets with oncogenic activity (e.g., Galectin 1 (*Lgals1*), Thrombospondin 1 (*Tsp1*)).

An enhanced glycolysis rate and fatty acid metabolism were extensively shown to support tumor initiation and progression [92,93]. A hyperactivation of glycolysis in HCC is indeed crucial to provide a rapid ATP energy source, but also for the production of intermediate metabolites feeding lipid, nucleotide, and amino acid synthesis to allow fast proliferation of cancerous cells [92,93]. Lipids are also important for the production of energy and membranes for rapidly proliferating cancer cells [94]. It is therefore likely that the deregulated metabolism characterizing the liver of miR22KO mice importantly contributes to the enhanced HCC development. In this regard, we identified four proteins involved in these pathways and upregulated in the liver of miR22KO mice (perilipin 2—*Plin2*, fatty acid binding protein 1—*Fabp1*, pyruvate kinase L/R—*Pklr*, and enolase 1—*Eno1*), whose expression is potentially regulated by miR-22. It is likely, however, that many other deregulated factors having their expressions normally fine-tuned or not by miR-22 also contribute to the increased glycolysis that we reported here and in our previous study [24]. As an example, Pyruvate kinase isoform M2 (*Pkm2*), a key promotor of glycolysis, but not a predicted miR-22 target, is also significantly upregulated in the liver of miR22KO mice [24]. Importantly, besides the roles of these proteins in hepatic glucose and lipid metabolism, most have been previously reported to also promote cancer in the liver and/or in other organs through mechanisms distinct from their metabolic functions. In this regard, almost all glycolytic enzymes favor carcinogenesis through a variety of metabolically unrelated mechanisms [95]. For example, PKM2, overexpressed in the liver of miR22KO mice, is a well-characterized tumor promotor affecting cancer progression and metastasis by regulating cell migration, angiogenesis, and stemness [96]. ENO1, in addition to deregulating the bioenergetics of cancer cells, was reported to sustain cell proliferation, to induce resistance to death, and to promote invasion and metastasis, in addition to angiogenesis [97]. Regarding the lipid metabolism, an upregulation of FABP1 in HCC was shown to stimulate angiogenesis and cancer cell migration [98], while PLIN2 overexpression is observed in many cancers and is suggested to have a role in tumorigenesis, for example, by favoring adaptation to hypoxia [99].

Unexpectedly, synthetic nucleotides mimicking the overexpression of miR-22 (miR-22 mimics) in human Huh7 hepatic cancer cells, or immortalized mouse AML12 hepatic cells, also promoted glycolysis, while leading to a striking decrease in lipid-dependent mitochondrial respiration. Although we do not have a clear explanation for the increased basal glycolytic rate in miR-22 overexpressing cancer cells, miR-22 re-expression in transformed cultured cancer cells, which have already undergone important metabolic switches, may have a different metabolic impact than miR-22 in wild-type primary hepatocytes within an orthotopic context. Our in vitro data remain relevant as they further outline the deep impact of miR-22 expression and activity on glucose metabolism and mitochondrial function. However, they also point to important differences in hepatic miR-22 functions in vivo and in vitro in transformed cancer cells, thus calling for caution when interpreting data. Since in our study miR-22 deletion seems to affect mostly tumor initiation, we evaluated the expression of potential targets of miR-22 in the non-tumoral part of the mouse livers expressing or not miR-22, rather than in tumors. Of note, DEN-induced tumors in WT mice fed either a CD or HFD have a decreased miR-22 expression, as observed in human HCC samples (Figure S5). In hepatocytes, the impact of miR-22 deletion or downregulation may also be completely different than the outcomes of miR-22 overexpression in cancer cells. Supporting this concept, the expressions of suggested miR-22 targets that were previously characterized in vitro in cancer cells were not affected by miR-22 deletion in hepatic mouse tissues, regardless of whether they were previously identified as tumor suppressors, e.g., PTEN or ERα, or as oncogenes, e.g., SIRT1, EZR, CD147, or SP1 [27,32,34,35,36,37,73] (Figure S9).

Among all non-metabolic potential miR-22 targets uncovered by our proteomic analysis, we showed that thrombospondin 1 (TSP1) is a direct target of miR-22 in hepatic cells and that this factor is consistently upregulated in miR-22-deficient hepatic tissues and downregulated in miR-22-overexpressing hepatic cancer cells. Several studies focusing on cancers other than that of the liver have already suggested a role for TSP1 upregulation in carcinogenesis, although this role still remains controversial [100,101,102]. TSP1 was indeed suggested to modulate proliferation and angiogenesis in cancer, but its role in HCC remains debated, and whether TSP1 behaves as a tumor suppressor or oncogene in the liver is still unclear [102,103]. TSP1 was reported as a marker of advanced HCC stages and to significantly correlate with vascular endothelial growth factor (VEGF) expression and venous invasion in HCC cancer cells [102]. However, in lymphoma and renal cell carcinoma, TSP1 was shown to display a clear antiangiogenic effect and TSP1-mimetic drugs are even being considered as potential anti-angiogenic therapeutics [104]. Finally, TSP1 can also be secreted in the blood and thus represents a potential biomarker [105]. Surprisingly, however, serum TSP1 in patients with HCC is significantly decreased, in contrast to levels reported for other human cancers, such as colorectal and lung carcinoma [105]. Based on these fragmentary data on HCC and other cancer types, it is thus difficult to delineate the role of TSP1 in normal and transformed hepatocytes. Our results with HFD-fed mice and bioinformatic analyses of the GEO dataset are in agreement with previous studies reporting an upregulation of TSP1 with hepatic steatosis [106]. Our in vitro data also clearly indicated that TSP1 downregulation restrains growth and migration of hepatic cancer cells, in particular in a NAFLD context (Figure 6). Together, these data support an oncogenic role for TSP1 in hepatocytes. However, in our in vitro conditions, cancer cells are not in contact with other hepatic stromal cells also expressing SP1 [102], and complex cell–cell interactions occurring in the tumoral microenvironment are absent. These data can therefore not be used to firmly conclude that TSP1 has oncogenic activity in the liver.

Altogether, our observations suggest that an administration of pharmacological agonists of miR-22, e.g., synthetic nucleotides mimicking miR-22, may have the potential to restrain HCC development and aggressiveness. However, our data also indicate that the role and function of miR-22 are highly dependent on the cell type and the pathophysiological context, thus calling for cautiousness when envisaging such therapeutic approaches. The specific targeting of hepatocytes, or cancer cells, in vivo with miR-22-mimicking pharmaceutical compounds, further remains an important technical challenge that has still not been overcome, but which needs to be resolved in order to prevent unwanted side effects of miR-22 mimics on non-hepatocytes cells [16]. The impact of miR-22 on hepatic immune cells, which are key factors modulating carcinogenesis [107], is, for example, still poorly understood. Finally, we did not consider in our study a potential role for the passenger strand miR-22-5p, which is also deleted in our miR-22KO mice. MicroRNA passenger strands are usually rapidly degraded and considered to not have any pathophysiological role in cells. However, this dogma has been recently challenged by showing that passenger strands can also, in some conditions, target specific mRNAs [108]. Future studies are now required to investigate these additional molecular aspects and the therapeutic impact of miR-22-3p synthetic nucleotides mimicking miR-22 on HCC development. It was indeed clearly demonstrated that an administration of synthetic nucleotides inhibiting specific microRNAs (e.g., miR-21) may have more distinct effects than the genetic deletion of the same microRNAs [109]. Whether synthetic nucleotide-mimicking miRNAs can be engineered to be highly stable, specifically targeted to hepatocytes and effective in vivo, is the next technical challenge to be overcome in this field of research.

## Data Availability

The data presented in this study are openly available in the Yareta data repository at the following https://doi.org/10.26037/yareta:5h2cavjznjclbj5b2iyvvjfkpi.

## Supplementary Materials

The following supporting information can be downloaded at: https://www.mdpi.com/article/10.3390/cells11182860/s1, Figure S1: Correlation of expression of miR22HG vs. β-catenin or p53 in HCC samples. Figure S2: Metabolic parameters of miR-22KO and WT mice. Figure S3: Albumin, α–antitrypsin, and Glypican3 expressions in WT and miR-22KO tumors. Figure S4: Long-chain fatty acid oxidation stress test in Huh7 cells overexpressing miR-22. Figure S5: MiR-22 expression in livers and tumors of DEN-injected WT mice. Figure S6: TSP1 expression in hepatic cell lines. Figure S7: 3′-UTR sequence of the TSP1 gene and mutation strategy of the miR-22 binding sites in the ′-UTR sequence of the TSP1. Figure S8: Regulation of cell proliferation and oncogene expression by miR-22. Figure S9: Expression of previously published target genes of miR-22 in the livers of miR22KO mice. Table S1: RT-qPCR primer list. Table S2: GEO dataset information. Table S3: Gene Ontology biological process enrichment analysis of upregulated proteins. Table S4: Enrichment analysis (String database). Table S5: Gene Ontology biological process enrichment analysis of downregulated proteins. Table S6: Identification of miR-22 targets involved in hepatocarcinogenesis.
