# Supplementary material for: MiR-22 Deficiency Fosters Hepatocellular Carcinoma Development in Fatty Liver
Source: Cells. 2022 Sep 14;11(18):2860. doi: 10.3390/cells11182860 (PMC9496902; doi:10.3390/cells11182860)
Supplement: Supplementary file 1 [file cells-11-02860-s001.zip › cells-1874933-supplementary.pdf]

MiR-22 deficiency fosters hepatocellular carcinoma development in fatty liver

Supplementary figures and tables

A

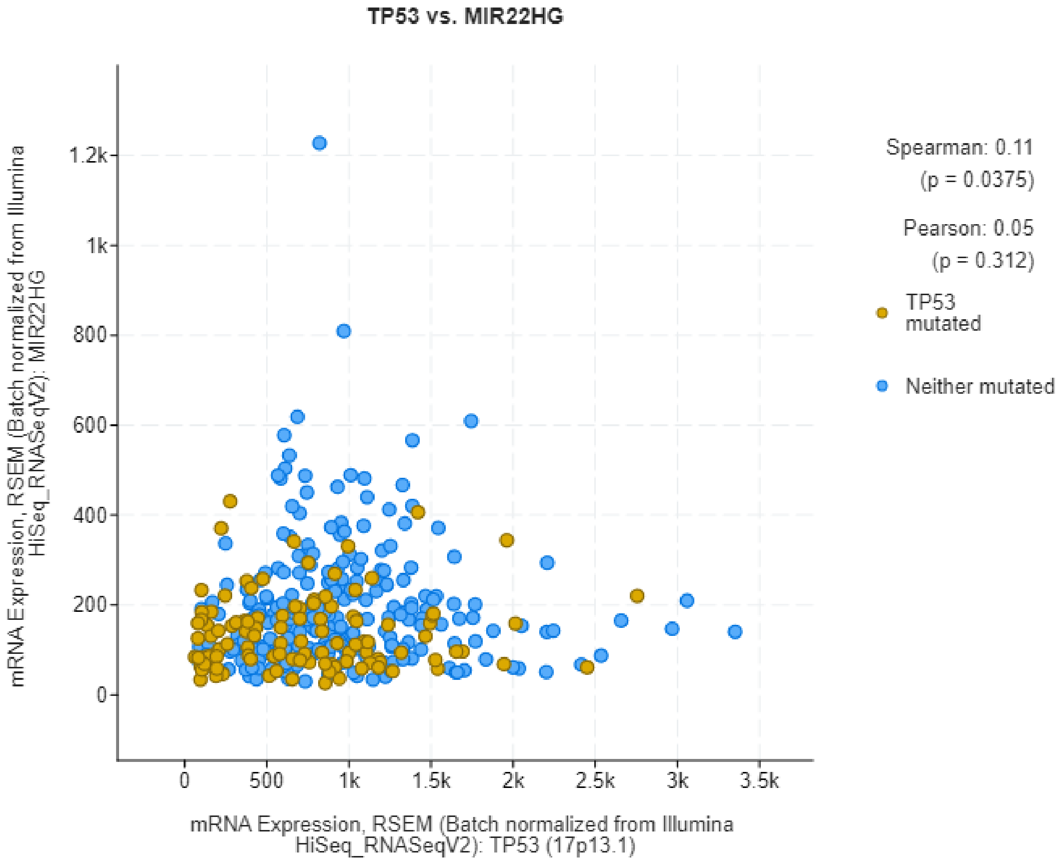

B

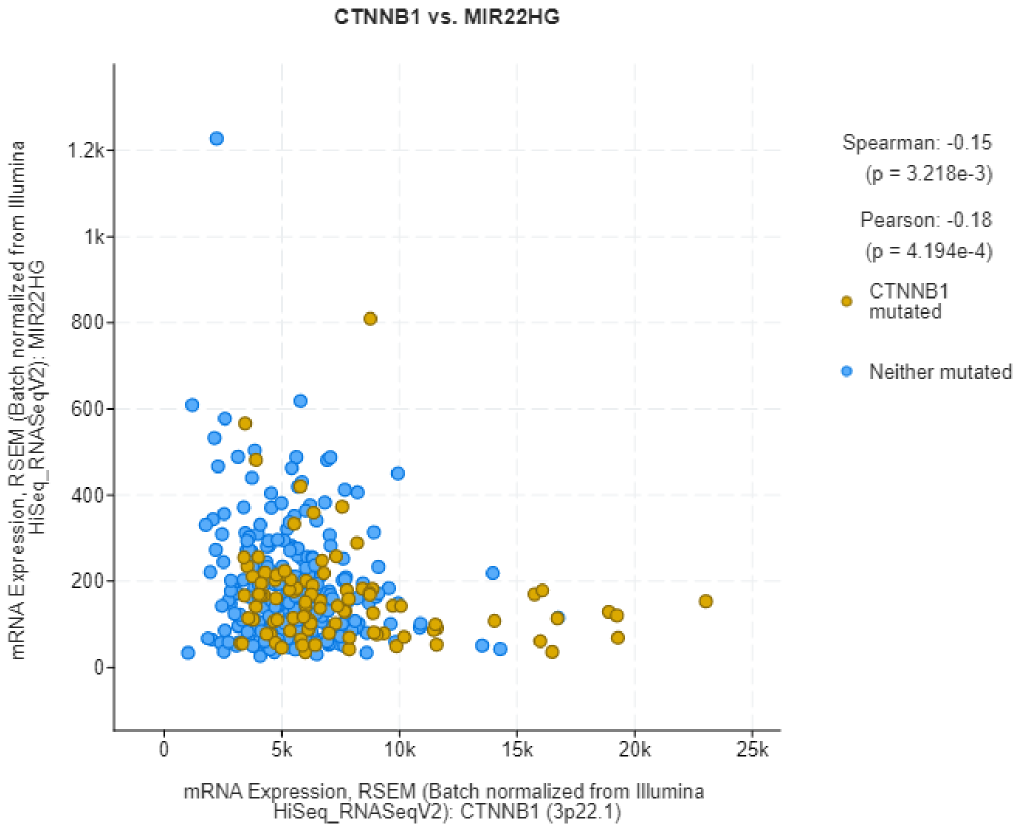

Supplementary Figure S1: Correlation of expression of miR22HG vs β-catenin or p53 in HCC samples.

(A) Correlation analysis of the RNA gene expression of *miR22HG* and p53 (*TP53*) in human HCC samples from the TCGA-LIHC cohort of patients. Samples from patients with a confirmed *TP53* mutation are marked in yellow, and patients without a *TP53* mutation are in blue.
(B) Correlation analysis of the RNA gene expression of *miR22HG* and β-catenin (*CTNNB1*) in human HCC samples from the TCGA-LIHC cohort of patients. Samples from patients with a confirmed *CTNNB1* mutation are marked in yellow, and patients without a *CTNNB1* mutation are in blue.
Graphical representation and statistical analysis (Spearman and Pearson test) were performed by the cBioPortal database (accessed 8 April 2022, https://www.cbioportal.org/).

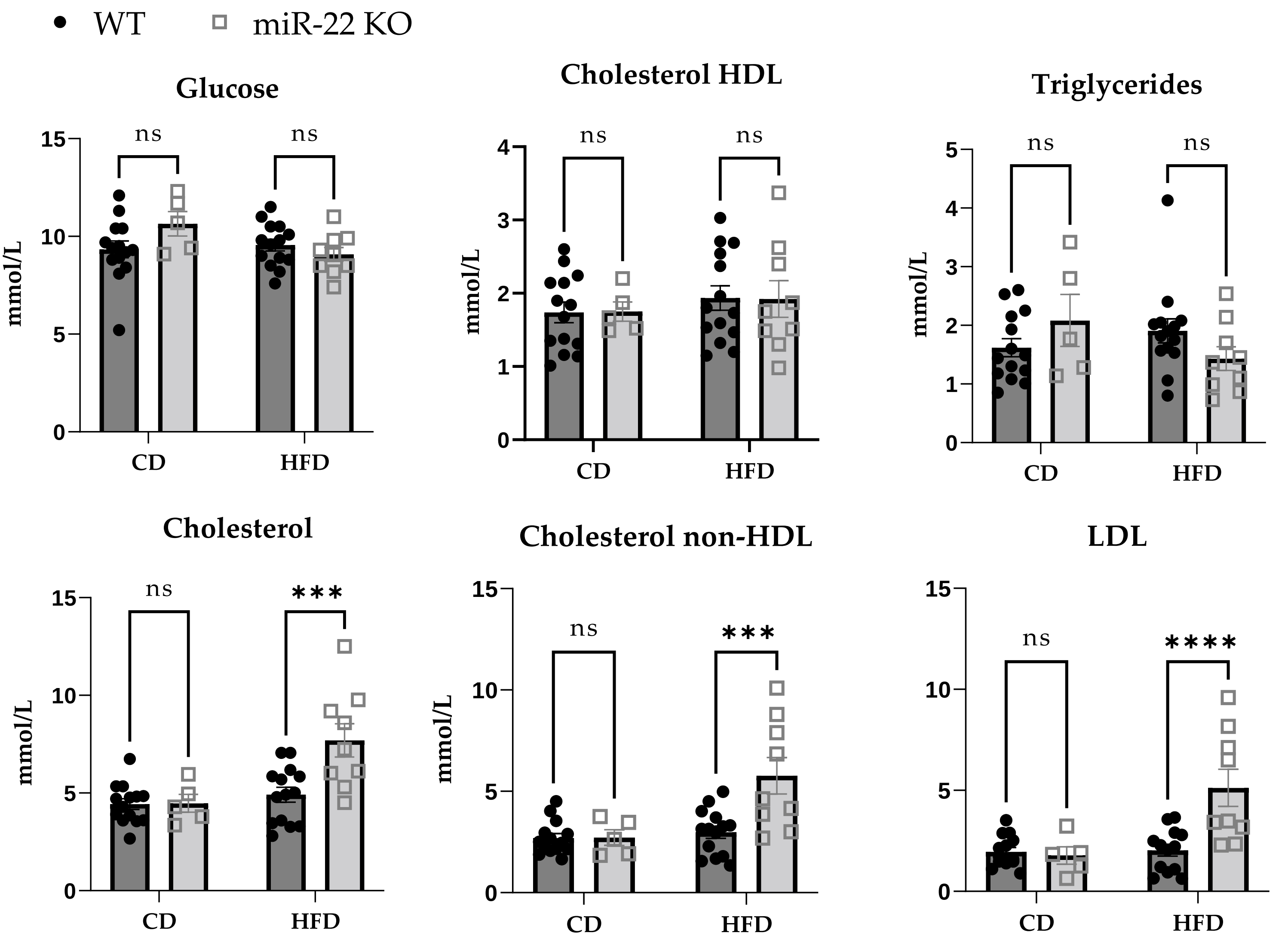

Supplementary Figure S2: Metabolic parameters of miR-22KO and WT mice.

Blood levels of glucose, cholesterol HDL, triglycerides, total cholesterol, non-HDL cholesterol and LDL in miR-22KO mice and WT littermates under standard chow diet (CD) or high-fat diet (HFD), at time of sacrifice. N=5-14/group.
Data is represented as mean +/- SEM. Two way ANOVA (multiple comparisons) were performed. P-values were represented as follows: *** $p \leq 0.001$; **** $p \leq 0.0001$.
ns – not significant ($p > 0.05$).

A

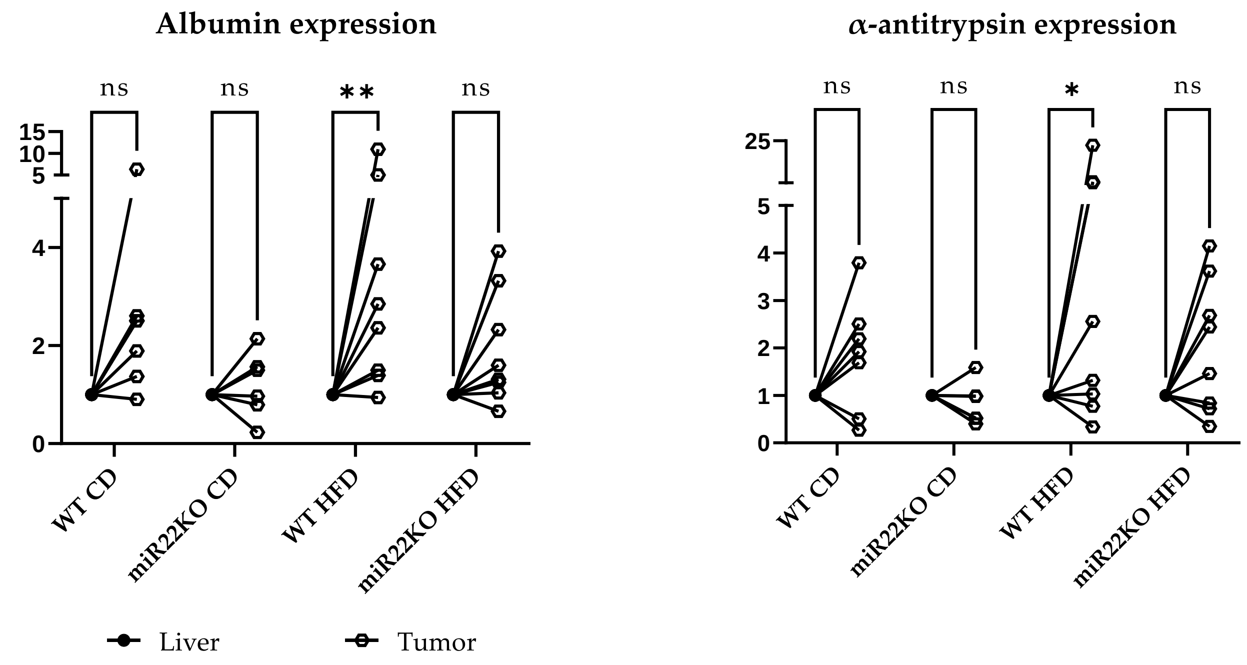

B

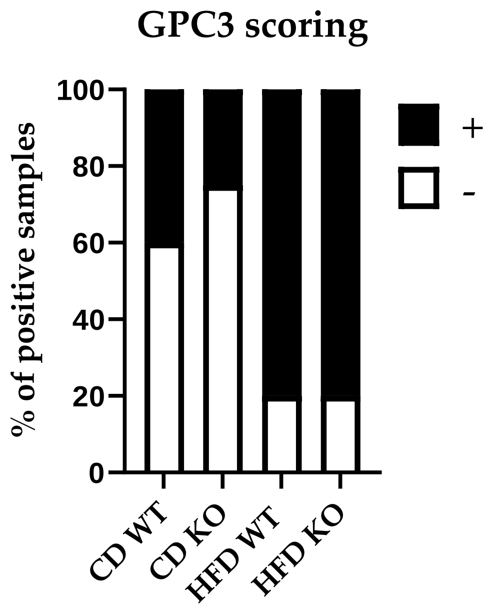

Supplementary Figure S3: Albumin, α–antitrypsin and Glypican3 expressions in WT and miR-22KO tumors.

(A) Albumin and α–antitrypsin (hepatic differentiation markers) mRNA expression was assessed via RT-qPCR in non-tumoral livers and associated tumors of miR-22KO mice and WT littermates under standard chow diet (CD) or high-fat diet (HFD), at time of sacrifice (n=6-8/group). The expression in the tumor was reported as a fold change in regards to the non-tumoral hepatic tissue of each mouse. Two way ANOVA (multiple comparisons) were performed. P- values were represented as follows: * $p \leq 0.05$; ** $p \leq 0.01$.
(B) Glypican 3 (*GPC3*) (HCC biomarker) mRNA expression was assessed via RT-qPCR in tumors of miR-22KO mice and WT littermates under standard chow diet (CD) or high-fat diet (HFD), at time of sacrifice (n=4-5/group). Samples with detectable levels of glypican 3 are marked as (+) and samples with non-detectable levels are marked with (-).
ns – not significant ($p > 0.05$).

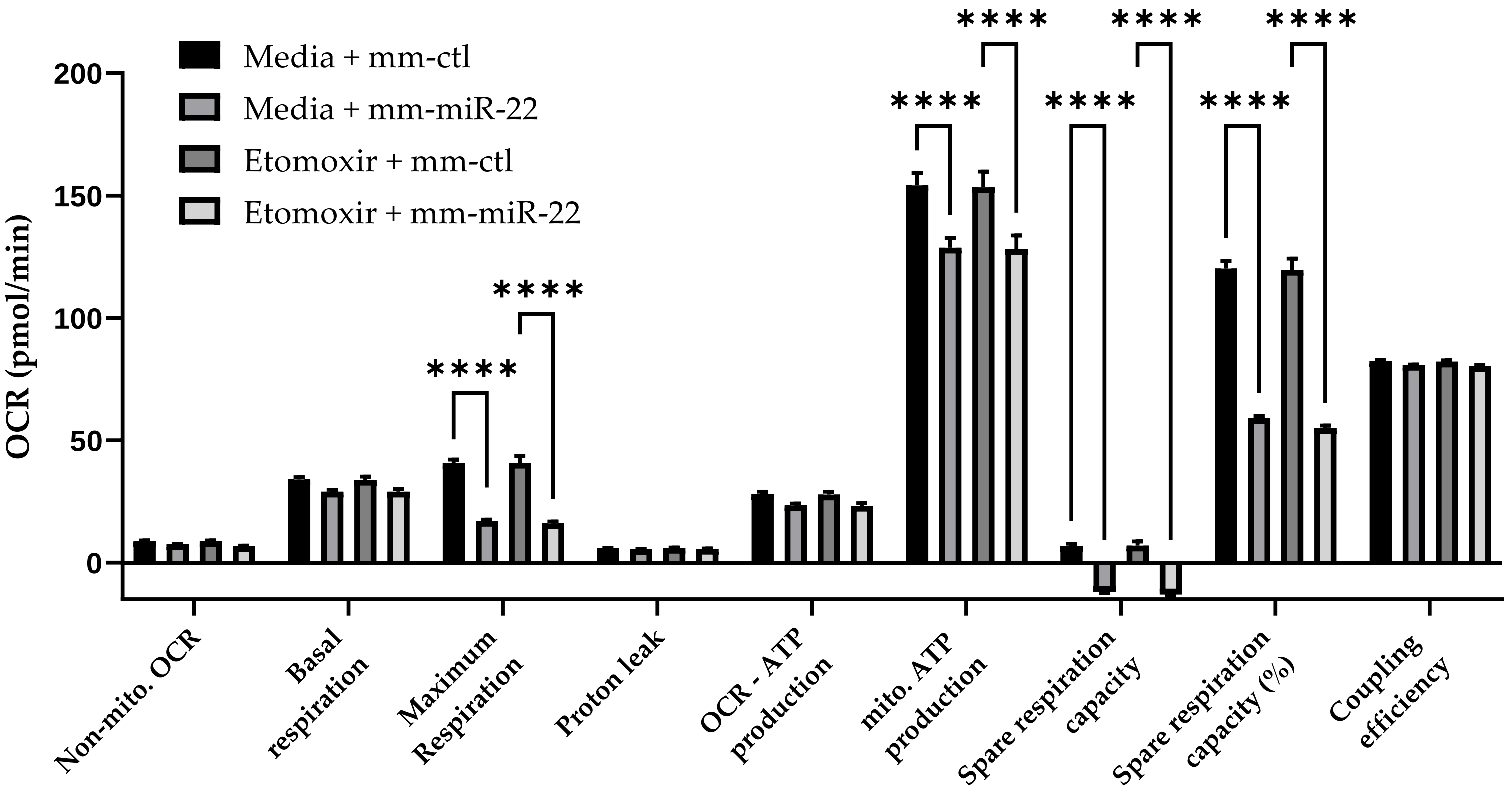

Supplementary Figure S4: Long-chain fatty acid oxidation stress test in Huh7 cells overexpressing miR-22.

Huh7 cells were transfected with 20nM oligonucleotides mimicking miR-22-3p (mm-miR-22-3p) or control oligonucleotides (mm-ctl), and 48h post-transfection, long chain fatty acid oxidation stress test (n=3) was performed (Seahorse Agilent protocol). Oxygen consumption rates (OCR) represented in Fig.5E were used to determine the non-mitochondrial OCR, basal respiration, maximum respiration, proton leak, ATP production, spare respiration capacity and coupling efficiency in these conditions.
Two way ANOVA (multiple comparisons) were performed. P- values were represented as follows: **** $p \leq 0.0001$.

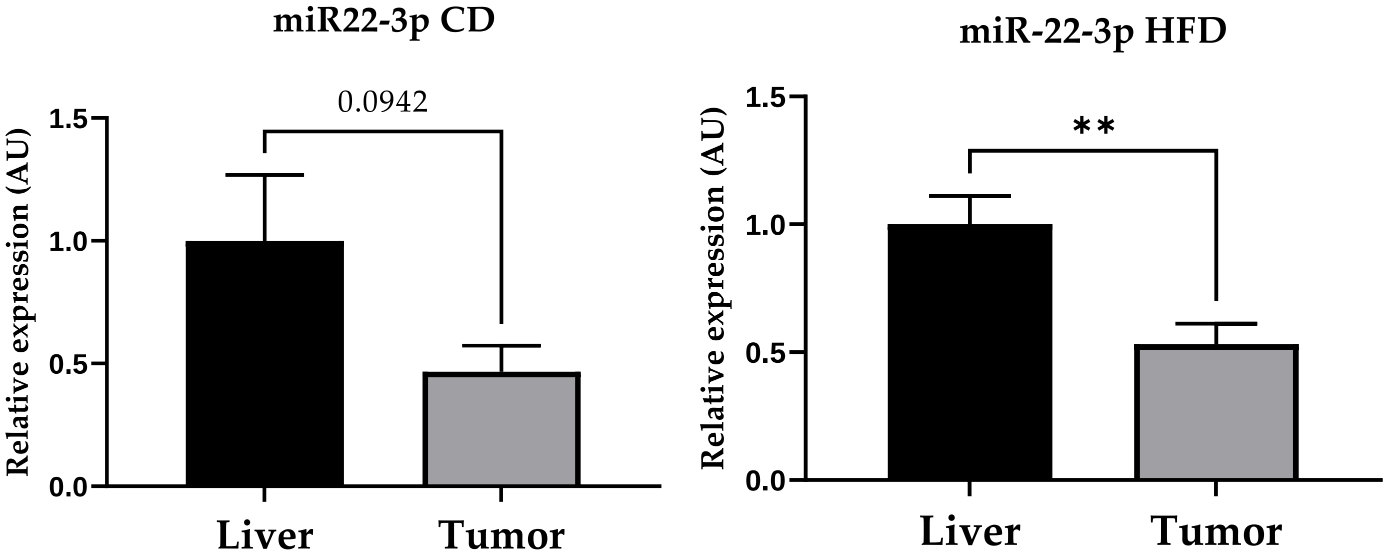

Supplementary Figure S5: MiR-22 expression in livers and tumors of DEN-injected WT mice.

MiR-22-3p expression was assessed via RT-qPCR in non-tumoral livers and associated tumors of WT mice injected with DEN under standard chow diet (CD, n=6) or high-fat diet (HFD, n=8), at time of sacrifice.

Data is represented as fold change, mean +/- SEM. Unpaired student's T-tests were performed. P-values were represented as follows: ** $p \leq 0.01$.

A

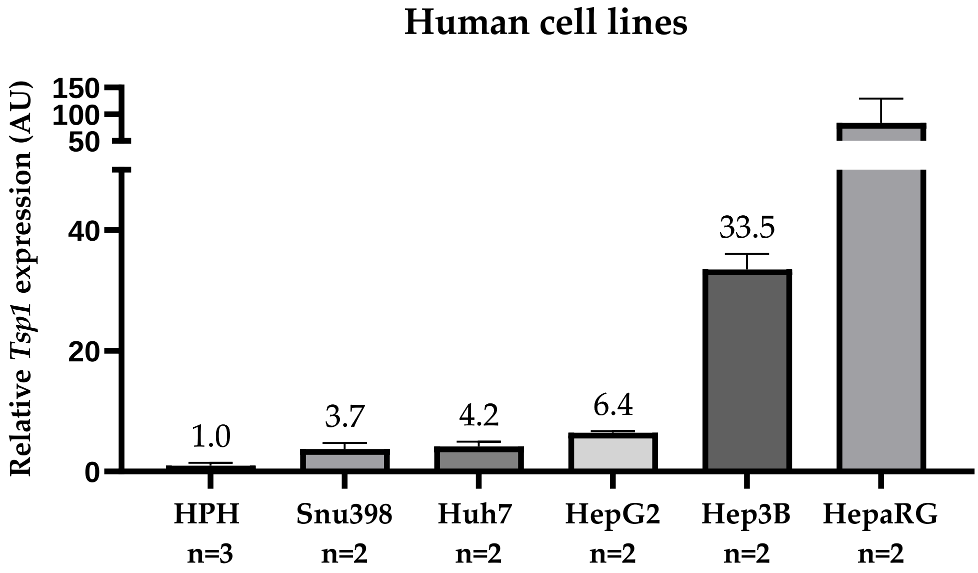

B

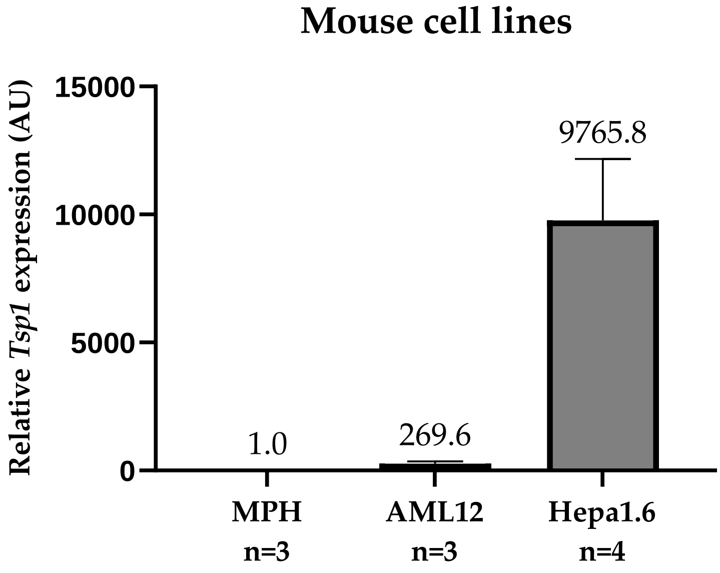

Supplementary Figure S6: TSP1 expression in hepatic cell lines.

(A) *TSP1* expression was assessed via RT-qPCR in human primary hepatocytes (HPH) and different immortalized / transformed human hepatic cell lines (Snu398, Huh7, HepG2, Hep3B, HepaRG).
(B) *Tsp1* expression was assessed via RT-qPCR in mouse primary hepatocytes (MPH) and different immortalized / transformed mouse hepatic cell lines (AML12, Hepa1.6).

Data is represented as fold change, mean +/- SEM.

A

TTGAAAGCCTTTGGAAAGCATAATATATGTTCTGGAAGGTTCACGCTGTGTCGGTCTCCTAGCATCAATGTCAGCTAATAAAATTAAATG
CTAATGTGCTTGAACAACCTTAAAATTAGGCTTTTGTCATTAGAAAAGTAGAGCTATTCCTATGTGGTTAACTTATTAACTAAGATGTCTA
TGCTTTTATGAATTAGTTTTCATTTGTATATTTATTTATATTTGTTTATTTAACAGATCCCTAATCATCAAATTGTTGATTGAAAGACTGATC
ATAAACCAATGCTGGTATTGCACCTTCTGGAACTATGGGCTTGAGAAAACCCCCAGGATCACTTCTCCTTGGCTTCCTTCTTTTCTGTGCTT
GCATCAGTGTGGACTCCTAGAACGTGCGACCTGCCTCAAGAAAATGCAGTTTTCAAAAACAGACTCAGCATTCAGCCTCCAATGAATAA
GACATCTTCCAAGCATATAAACAATTGCTTTGGTTTCCTTTTGAAAAAGCATCTACTTGCTTCAGTTGGGAAGGTGCCCATTCCACTCTGC
CTTTGTCACAGAGCAGGGTGCTATTGTGAGGCCATCTCTGAGCAGTGGACTCAAAAGCATTTTCAGGCATGTCAGAGAAGGGAGGACTC
ACTAGAATTAGCAAACAAAACCACCCTGACATCCTCCTTCAGGAACACGGGGAGCAGAGGCCAAAGCACTAAGGGGAGGGCGCATAC
CCGAGACGATTGTATGAAGAAAATATGGAGGAACTGTTACATGTTCGGTACTAAGTCATTTTCAGGGGATTGAAAGACTATTGCTGGATT
TCATGATGCTGACTGGCGTTAGCTGATTAACCCATGTAAATAGGCACTTAAATAGAAGCAGGAAAGGGAGACAAAGACTGGCTTCTGGA
CTTCCTCCCTGATCCCCACCCTTACTCATCACCTGCAGTGGCCAGAATTAGGGAATCAGAATCAAACCAGTGTAAGGCAGTGCTGGCTGC
CATTGCCTGGTCACATTGAAATTGGTGGCTTCATTCTAGATGTAGCTTGTGCAGATGTAGCAGGAAAATAGGAAAACCTACCATCTCAGT
GAGCACCAGCTGCCTCCCAAAGGAGGGGCAGCCGTGCTTATATTTTTATGGTTACAATGGCACAAAATTATTATCAACCTAACTAAAACA
TTCCTTTTCTCTTTTTTCCTGAATTATCATGGAGTTTTCTAATTCTCTCTTTTGGAATGTAGATTTTTTTTAAATGCTTTACGATGTAAAATATT
TATTTTTTACTTATTCTGGAAGATCTGGCTGAAGGATTATTCATGGAACAGGAAGAAGCGTAAAGACTATCCATGTCATCTTTGTTGAGAG
TCTTCGTGACTGTAAGATTGTAAATACAGATTATTTATTAACTCTGTTCTGCCTGGAAATTTAGGCTTCATACGGAAAGTGTTTGAGAGCA
AGTAGTTGACATTTATCAGCAAATCTCTTGCAAGAACAGCACAAGGAAAATCAGTCTAATAAGCTGCTCTGCCCCTTGTGCTCAGAGTGG
ATGTTATGGGATTCTTTTTTTCTCTGTTTTATCTTTTCAAGTGGAATTAGTTGGTTATCCATTTGCAAATGTTTTAAATTGCAAAGAAAGCCA
TGAGGTCTTCAATACTGTTTTACCCCATCCCTTGTGCATATTTCCAGGGAGAAGGAAAGCATATACACTTTTTTCTTTCATTTTTCCAAAAG
AGAAAAAAATGACAAAAGGTGAAACTTACATACAAATATTACCTCATTTGTTGTGTGACTGAGTAAAGAATTTTTGGATCAAGCGGAAA
GAGTTTAAGTGTCTAACAAACTTAAAGCTACTGTAGTACCTAAAAAGTCAGTGTTGTACATAGCATAAAAACTCTGCAGAGAAGTATTCC
CAATAAGGAAATAGCATTGAAATGTTAAATACAATTTCTGAAAGTTATGTTTTTTTTCTATCATCTGGTATACCATTGCTTTATTTTTATAA
ATTATTTTCTCATTGCCATTGGAATAGATATCTCAGATTGTGTAGATATGCTATTTAAATAATTTATCAGGAAATACTGCCTGTAGAGTTAG
TATTTCTATTTTTATATAATGTTTGCACACTGAATTGAAGAATTGTTGGTTTTTTCTTTTTTTTGTTTTGTTTTTTTTTTTTTTTTTTTTGCTTTTG
ACCTCCCATTTTTACTATTTGCCAATACCTTTTTCTAGGAATGTGCTTTTTTTTGTACACATTTTTATCCATTTTACATTCTAAAGCAGTGTAA
GTTGTATATTACTGTTTCTTATGTACAAGGAACAACAATAAATCATATGGAAATTTATATTTATACTTACTGTATCCATGCTTATTTGTTCTC
TACTGGC

B

Mutated site	Forward primer	Reverse primer
3`-UTR-TSP1-mut1	ATCACTTCTCCTTGGCTTCCGTCGACTTCTGTGCTTGCATCAGTGT	ACACTGATGCAAGCACAGAAGTCGACGGAAGCCAAGGAGAAGTGAT
3`-UTR-TSP1-mut2	GCTGGATTTCATGATGCTGAGTCGACGTTAGCTGATTAACCCATGT	ACATGGGTTATCAGCTAACGTCGACTCAGCATCATGAAATCCAGC
3`-UTR-TSP1-mut3	AATTGGTGGCTTCTAGATGTGTCGACGTGCAGATGTAGCAGGAAAA	TTTTCCTGCTACATCTGCACGTCGACACATCTAGAAGCCACCAATT

C

WT site 1 ATCACTTCTCCTTGGCTTCC--T-T-C-T-T-T-----TCTGTGCTTGCATCAGTG
Mut site 1 ATCACTTCTCCTTGGCTTCC-G-T-C-G-A-C-T-TCTGTGCTTGCATCAGTG

WT site 2 GCTGGATTTCATGATGCTGA---C-T-G-G--CGTTAGCTGATTAACCCATGT
Mut site 2 GCTGGATTTCATGATGCTGA-G-T-C-G-A-CGTTAGCTGATTAACCCATGT

WT site 3 AATTGGTGGCTTC-A-T-T-C-T-A-GA-T-G-T-A-G-C-T-T-GTGCAGATGTAGCAGGAAAA
Mut site 3 AATTGGTGGCTTC----T--A-G-A-T-G-T-G-T-C-G-A--C---GTGCAGATGTAGCAGGAAAA

Supplementary Figure S7: 3`-UTR sequence of the TSP1 gene and mutation strategy of the miR-22 binding sites.

(A) Genetic sequence of the 3`-UTR of the human TSP1 gene that was cloned in a LightSwitch 3´-UTR Reporter Go Clone vector and thus fused with the luciferase reporter gene. This vector was used to determine the binding capacity of miR-22 to the 3`-UTR of the TSP1 transcript. The 3 putative miR-22-3p binding sites that were mutated are marked in red and the primers used to mutate the sites are marked in yellow, green and purple (Mut1, Mut2 and Mut3, respectively).
(B) Forward and reverse primers used to create Mut1, Mut2 and Mut3.
(C) Representative alignment of the WT sites and the newly created mutated sites. Differences are highlighted in red.

A

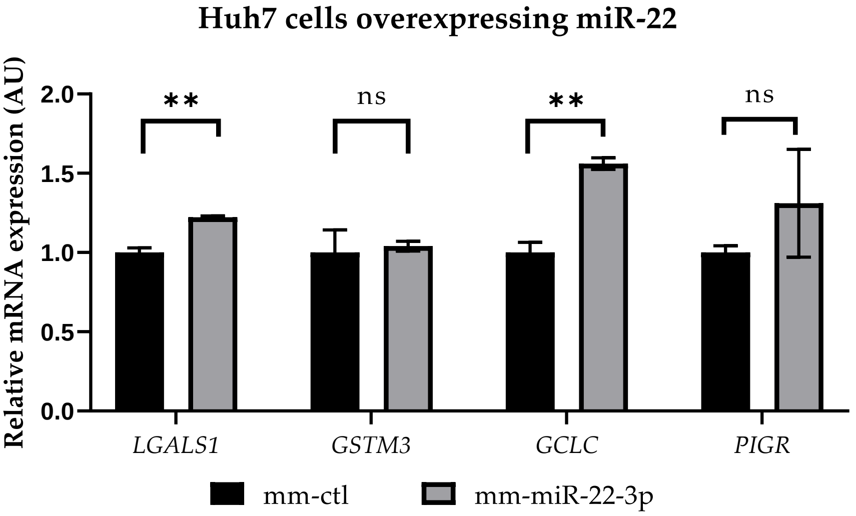

B

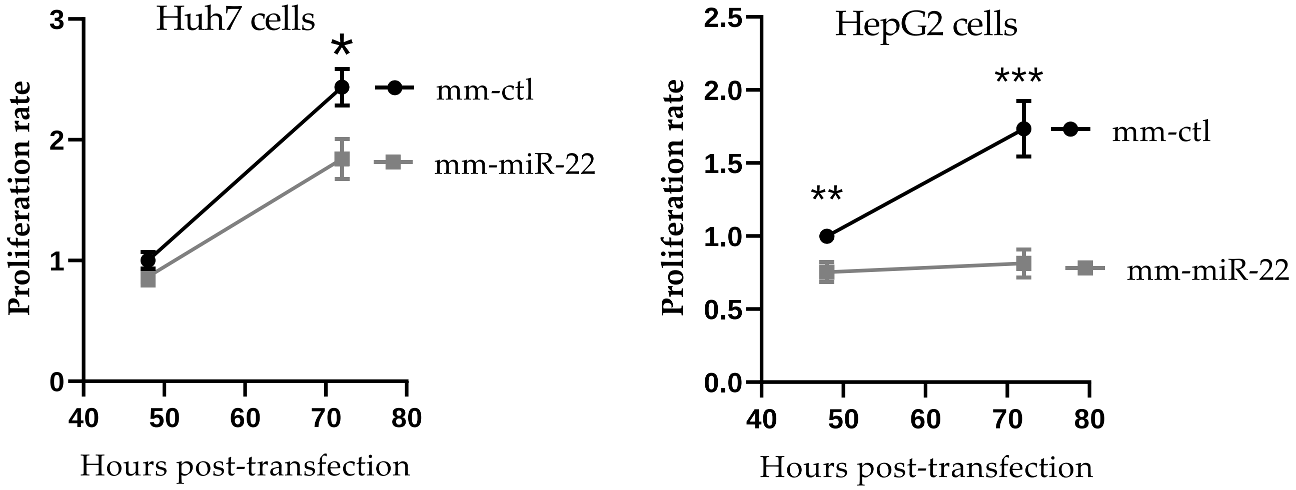

Supplementary Figure S8: Regulation of cell proliferation and oncogene expression by miR-22.

(A) RT-qPCR analysis of mRNA expression of *LGALS1, GSTM3, GCLC* and *PIGR* in Huh7 cells transfected with miR-22-3p mimicking oligo-nucleotides (mm-miR-22-3p) or control oligonucleotides (mm-ctl), 48h post-transfection (n=3).
(B) Proliferation rate of Huh7 and HepG2 cells transfected with miR-22-3p mimicking oligo-nucleotides (mm-miR-22-3p) or control oligonucleotides (mm-ctl), 48h and 72h post-transfection.
Data is represented as fold change, mean +/- SEM. Unpaired student's T-tests were performed. P-values were represented as follows: * $p \leq 0.05$; ** $p \leq 0.01$; *** $p \leq 0.001$.
ns – not significant ($p > 0.05$).

Tumor suppressors

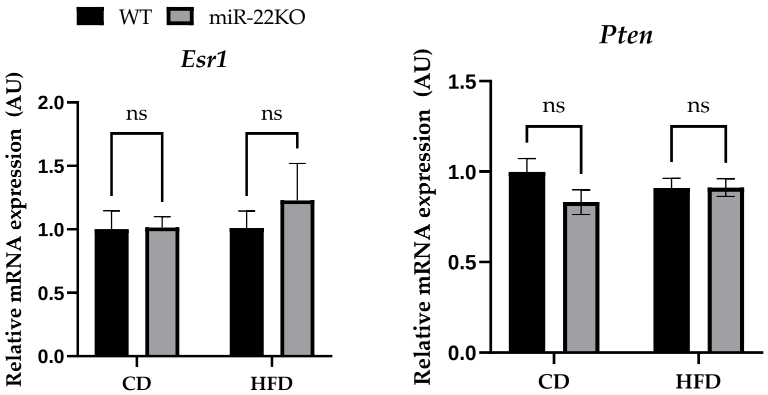

Oncogenes

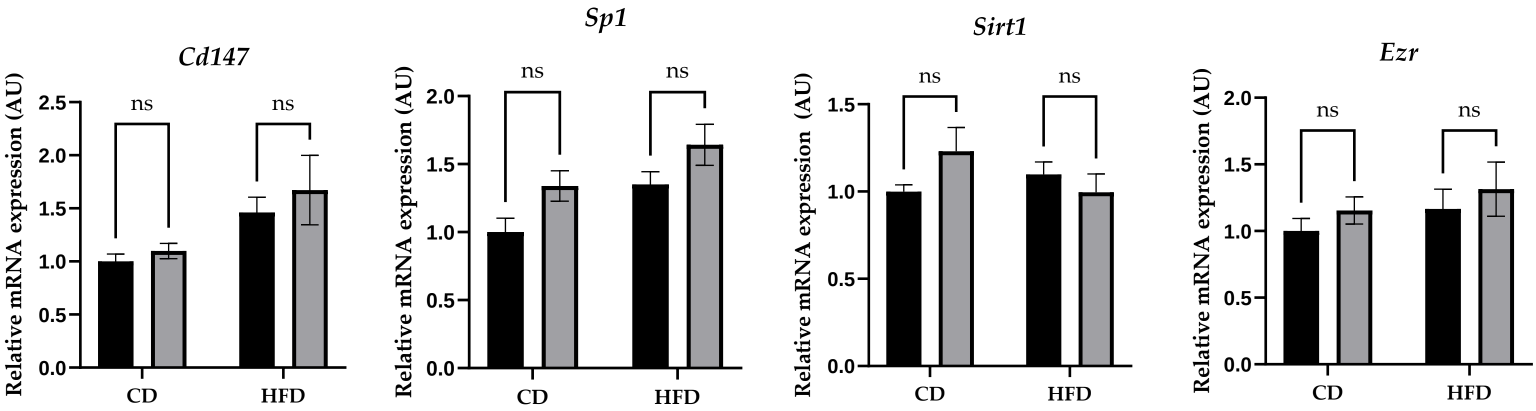

Supplementary Figure S9: Expression of previously published target genes of miR-22 in the livers of miR22KO mice.

RT-qPCR analysis of mRNA expression of *Esr1, Pten, Cd147, Sp1, Sirt1* and *Ezr* in the livers of DEN-treated miR-22KO and WT mice under CD or HFD for 9 months (diet began at 2 months of age, n=5-9 mice / group).
Data is represented as fold change, mean +/- SEM and two-way ANOVA (multiple comparison test) were performed (ns – not significant, $p > 0.05$).

Mouse primers	
**Primer name**	**Primer sequence**
**mm-Eno1 fw**	AGT ACG GGA AGG ACG CCA CCA
**mm-Eno1 rev**	GCG GCC ACA TCC ATG CCG AT
**mm-Pklr fw**	CTT TGC CTC CTT TGT ACG A
**mm-Pklr rev**	TCA TCA AAC TTC TTC ACG CC
**mm-Fabp1 fw**	AAA GTC AAG GCA GTC GTC
**mm-Fabp1 rev**	CCC AAT GTC ATG GTA TTG GT
**mm-Plin2 fw**	TAA TGC CAT CAC CAA GTC GG
**mm-Plin2 rev**	CTT CCA TCT CCA GCT CCT C
**mm-Tsp1 fw**	AAT CAT GGC TGA CTC GGG AC
**mm-Tsp1 rev**	GCG CTG GTT ATG ATT GGC AG
**mm-Lgals1 fw**	ATG GAG ACG CCA ACA CCA
**mm-Lgals1 rev**	AGC TTG ATG GTC AGG TCA
**mm-Gstm3 fw**	CCC CAA CTT TGA CCG AAG C
**mm-Gstm3 rev**	GGT GTC CAT AAC TTG GTT CTC CA
**mm-Gclc fw**	CCC AAT TGT TAT GGC TTT GAG
**mm-Gclc rev**	GAG ACT TAC TGA TCC TAA AGC G
**mm-Hpgd fw**	CAA CAA TGC AGG CGT GAA CA
**mm-Hpgd rev**	GCG TGT GAA TCC GAT GAT GC
**mm-Pigr fw**	ATG AGG CTC TAC TTG TTC ACG C
**mm-Pigr rev**	ACC TCC TGG GGA CCA AAT ATG
**mm-Alb fw**	TGC TTT TTC CAG GGG TGT GTT
**mm-Alb rev**	TTA CTT CCT GCA CTA ATT TGG CA
**mm-Antitryp fw**	CCC GGA TCT TCA ACA ATG G
**mm-Antitryp rev**	TTA TGC ACA GCC TTG CTG
**mm-Gpc3 fw**	CAG CCC GGA CTC AAA TGG G
**mm-Gpc3 rev**	GCC GTG CTG TTA GTT GGT ATT TT
**mm-Esr1 fw**	TGT GTC CAG CTA CAA ACC AAT G
**mm-Esr1 rev**	CAT CAT GCC CAC TTC GTA ACA
**mm-Pten fw**	ACA CCG CCA AAT TTA ACT GC
**mm-Pten rev**	TAC ACC AGT CCG TCC CTT TC
**mm-Cd147 fw**	GTG GCG TTG ACA TCG TTG G
**mm-Cd147 rev**	CTA TGT ACT TCG TAT GCA GGT CG
**mm-Sp1 fw**	AGG GTC CGA GTC AGT CAG G
**mm-Sp1 rev**	CTC GCT GCC ATT GGT ACT GTT
**mm-Sirt1 fw**	AGT TCC AGC CGT CTC TGT GT
**mm-Sirt1 rev**	CTC CAC GAA CAG CTT CAC AA
**mm-Ezr fw**	CAA TCA ACG TCC GGG TGA C
**mm-Ezr rev**	GCC AAT CGT CTT TAC CAC CTG A
**mm-CycloA fw**	CAA ATG CTG GAC CAA ACA CAA
**mm-CycloA rev**	GCC ATC CAG CCA TTC AGT CT

Human primers	
**Primer name**	**Primer sequence**
**hs-Tsp1 fw**	ATG GAG AAT GCT GTC CTC GC
**hs-Tsp1 rev**	CCA TTG CCA CAG CTC GTA GA
**hs-Lgals1 fw**	GGG CAC ATT AAA GGT GGC AT
**hs-Lgals1 rev**	TGC AAA GCT CTC TGG GTT GA
**hs-Gstm3 fw**	TCG TGC GAG TCG TCT ATG GT
**hs-Gstm3 rev**	TCT CCT CAT AAG AGG TAT CCG TG
**hs-Gclc fw**	GGT GAC ATT CCA AGC CTG
**hs-Gclc rev**	CTT CAA TGG CTC CAG TCC
**hs-Pigr fw**	AGT CCC ATA TTT GGT CCC GAG
**hs-Pigr rev**	AGG TGG GTG GGT AGT AGC AC
**hs-CycloA fw**	ATG GTC AAC CCC ACC GTG T
**hs-CycloA rev**	TCT GCT GTC TTT GGG ACC TTG TC

Supplementary Table S1: RT-qPCR primer list.

List of human and mouse primers used for RT-qPCR. Cyclophilin A (CycloA) was used as a housekeeping gene.

Human Hepatocellular carcinoma models				
**Dataset nb**	**Samples**	**Method**	**Study**	**PMID**
**GSE64041**	60 non-tumoral liver biopsies and 60 HCC biopsies	array	Gene expression profiling in paired human hepatocellular carcinoma and liver parenchyma biopsies and normal liver biopsies.	27499918
**GSE60502**	18 non-tumoral liver biopsies and 18 HCC biopsies	array	Gene expression profiling of 18 hepatocellular carcinoma and adjacent non-tumorous liver tissue	25376302
**GSE21362**	73 non-tumoral liver biopsies and 73 HCC biopsies	array	MicroRNA Profile Predicts Recurrence of Hepatocellular Carcinoma in Milan Criteria Cases with Mild Liver Cirrhosis.	21298008
**GSE10694**	78 non-tumoral liver biopsies and 78 HCC biopsies	array	MicroRNAs expression profile in human hepatocellular carcinoma	18649363
**GSE36915**	21 non-tumoral liver biopsies and 68 HCC biopsies	array	MicroRNA-214 downregulation contributes to tumor angiogenesis via inducing secretion of hepatoma-derived growth factor in human hepatoma	22613005
**Mouse hepatic cancer models**				
**Dataset nb**	**Samples**	**Method**	**Study**	**PMID**
**GSE26538**	6 HCC and 6 normal livers from B6C3F1 mice	array	Global gene expression profiling of spontaneous hepatocellular carcinoma in B6C3F1 mice: Similarities in the molecular landscape to human liver cancer.	21571946
**GSE31431**	5 PDGFC tumor samples and 4 normal liver samples of mice	array	Serial gene expression profiling in the liver of Pdgf-c Tg mice that developed hepatic fibrosis and tumors	22651928
**GSE63027**	4 GNMT knock-out HCC samples and 5 normal liver samples at 8 month age	array	Expression data from GNMT and MAT1A knockout models that develop all the stages of non-alcoholic fatty liver disease including hepatocellular carcinoma	25993042
**GSE19004**	5 Rb, p130 and p107 knock-out HCC samples and 4 normal liver samples	array	Mouse HCC model	ND
**GSE29813**	6 HCC and 6 normal livers from B6C3F1 mice	array	Global gene expression profiling of hepatocellular carcinomas in B6C3F1 mice induced by Ginkgo biloba extract by gavage for two years	23262642
**GSE102416**	5 DEN-induced tumors and 5 non-tumoral livers of WT mice	array	A comparative miRNA/mRNA analysis in distinct murine liver cancer models reveals miR-193a-5p and NUSAP1 as therapeutic targets in HCC [mRNA]	30165047
**GSE66717**	4 liver specific PTEN knock-out livers and 3 WT livers	array	Hepatocyte-specific knockout of Pten and of Pten and Tgfbr2 in mice	26627606

Supplementary Table S2: GEO dataset information.

Detailed list of GEO datasets containing microarray data of human HCC patient cohorts and mouse models of hepatocarcinogenesis used to assess the expression of miR-22, *miR22HG* or Thrombospondin-1.

Category	ID	term	Gene Ratio	Bg Ratio	pvalue	adj_pval	qvalue	genes	Count
GO:0006631	GO:0006631	fatty acid metabolic process	33/118	392/18862	3.06E-28	3.13E-25	2.32E-25	ABHD1/ACAT1/ACAT2/CRAT/ACOX1/HPGD/EHHADH/APOA4/ACACB/ACACA/ACAD11/ELOVL2/ACLY/ADH4/ELOVL5/FASN/PRKAG1/ALDH3A2/DECR2/ACSS2/MGLL/ACOT1/ACOT2/ACOT4/ACSL5/CD36/FADS6/GSTA1/FADS1/ABCD2/FABP1/FABP5/ACADM	33
GO:0044282	GO:0044282	small molecule catabolic process	34/118	431/18862	3.62E-28	3.13E-25	2.32E-25	ALDH1A1/GK/ENO1/ABHD1/SHMT1/ACAT1/ACAT2/TYMP/CRAT/UPB1/ACOX1/EHHADH/PGD/GPD2/ACACB/ACAD11/ADH4/SULT1A1/HNMT/ALDH3A2/DECR2/GAPDH/ACOT4/TKFC/BDH1/PKLR/ALDOB/ABCD2/AKR1D1/ASPA/FABP1/UROC1/ACADM/GCK	34
GO:0046394	GO:0046394	carboxylic acid biosynthetic process	25/118	327/18862	2.29E-20	1.32E-17	9.79E-18	ABHD1/SHMT1/UPB1/HPGD/APOA4/ACACB/ACACA/ELOVL2/ACLY/ELOVL5/FASN/PRKAG1/DECR2/ACSS2/MGST3/MGLL/ACOT4/PKLR/FADS6/FADS1/ABCD2/AKR1D1/ASPA/UGP2/FABP5	25
GO:0016053	GO:0016053	organic acid biosynthetic process	25/118	335/18862	4.13E-20	1.79E-17	1.32E-17	ABHD1/SHMT1/UPB1/HPGD/APOA4/ACACB/ACACA/ELOVL2/ACLY/ELOVL5/FASN/PRKAG1/DECR2/ACSS2/MGST3/MGLL/ACOT4/PKLR/FADS6/FADS1/ABCD2/AKR1D1/ASPA/UGP2/FABP5	25
GO:0072330	GO:0072330	monocarboxylic acid biosynthetic process	20/118	224/18862	9.69E-18	3.35E-15	2.48E-15	ABHD1/HPGD/APOA4/ACACB/ACACA/ELOVL2/ACLY/ELOVL5/FASN/PRKAG1/DECR2/ACSS2/MGLL/ACOT4/PKLR/FADS6/FADS1/ABCD2/AKR1D1/FABP5	20
GO:0006633	GO:0006633	fatty acid biosynthetic process	18/118	168/18862	1.79E-17	5.17E-15	3.83E-15	ABHD1/HPGD/APOA4/ACACB/ACACA/ELOVL2/ACLY/ELOVL5/FASN/PRKAG1/DECR2/ACSS2/MGLL/ACOT4/FADS6/FADS1/ABCD2/FABP5	18
GO:0046395	GO:0046395	carboxylic acid catabolic process	20/118	243/18862	4.82E-17	1.19E-14	8.81E-15	ABHD1/SHMT1/ACAT1/ACAT2/CRAT/ACOX1/EHHADH/PGD/ACACB/ACAD11/HNMT/ALDH3A2/DECR2/ACOT4/ABCD2/AKR1D1/ASPA/FABP1/UROC1/ACADM	20
GO:0016054	GO:0016054	organic acid catabolic process	20/118	258/18862	1.55E-16	3.36E-14	2.49E-14	ABHD1/SHMT1/ACAT1/ACAT2/CRAT/ACOX1/EHHADH/PGD/ACACB/ACAD11/HNMT/ALDH3A2/DECR2/ACOT4/ABCD2/AKR1D1/ASPA/FABP1/UROC1/ACADM	20
GO:0006637	GO:0006637	acyl-CoA metabolic process	14/118	105/18862	3.63E-15	5.78E-13	4.28E-13	ACAT1/ACACB/ACACA/ELOVL2/ACLY/ELOVL5/FASN/ACSS2/ACOT13/ACOT1/ACOT2/HMGCS2/ACOT4/ACSL5	14
GO:0035383	GO:0035383	thioester metabolic process	14/118	105/18862	3.63E-15	5.78E-13	4.28E-13	ACAT1/ACACB/ACACA/ELOVL2/ACLY/ELOVL5/FASN/ACSS2/ACOT13/ACOT1/ACOT2/HMGCS2/ACOT4/ACSL5	14

Supplementary Table S3: Gene Ontology biological process enrichment analysis of upregulated proteins.

148 proteins that were found significantly upregulated in the livers of miR-22KO mice at 3 months of HFD (Gjorgjieva et al.,JPM.2022) were submitted to an Gene ontology enrichment analysis of biological process *via* the R software packages GOdb and clusterProfiler. The 10 most significantly enriched processes were retained and presented as a chordplot in Fig.4C.

FDR	nGenes	GO terms or pathways	Description
0.0000	117	GO.0044281	small molecule metabolic process
0.0000	67	GO.0032787	monocarboxylic acid metabolic process
0.0000	79	GO.0019752	carboxylic acid metabolic process
0.0000	81	GO.0006082	organic acid metabolic process
0.0000	86	GO.0006629	lipid metabolic process
0.0000	82	GO.0055114	oxidation-reduction process
0.0000	67	GO.0044255	cellular lipid metabolic process
0.0000	51	GO.0051186	cofactor metabolic process
0.0000	44	GO.0006631	fatty acid metabolic process
0.0000	45	GO.0008610	lipid biosynthetic process
0.0000	48	GO.0044283	small molecule biosynthetic process
0.0000	214	GO.0008152	metabolic process
0.0000	37	GO.0006732	coenzyme metabolic process
0.0000	46	GO.0055086	nucleobase-containing small molecule metabolic process
0.0000	43	GO.0009117	nucleotide metabolic process
0.0000	32	GO.0046394	carboxylic acid biosynthetic process
0.0000	27	GO.0072330	monocarboxylic acid biosynthetic process
0.0000	52	GO.0019637	organophosphate metabolic process
0.0000	195	GO.0071704	organic substance metabolic process
0.0000	185	GO.0044237	cellular metabolic process
0.0000	34	GO.0019693	ribose phosphate metabolic process
0.0000	38	GO.0017144	drug metabolic process
0.0000	18	GO.0006637	acyl-CoA metabolic process
0.0000	32	GO.0009150	purine ribonucleotide metabolic process
0.0000	181	GO.0044238	primary metabolic process
0.0000	33	GO.0072521	purine-containing compound metabolic process
0.0000	27	GO.0006790	sulfur compound metabolic process
0.0000	60	GO.0044248	cellular catabolic process
0.0000	24	GO.0008202	steroid metabolic process
0.0000	18	GO.0006694	steroid biosynthetic process

Supplementary Table S4: Enrichment analysis (String database).

148 proteins that were found significantly upregulated in the livers of miR-22KO mice at 3 months of HFD (Gjorgjieva et al.,JPM.2022) were submitted to an Gene ontology enrichment analysis of process *via* the STRING database (https://string-db.org/). The 30 most significantly enriched processes are represented above.

Category	ID	term	GeneRatio	BgRatio	pvalue	adj_pval	qvalue	genes	Count
GO:0016125	GO:0016125	sterol metabolic process	12/149	165/18862	7.57E-09	1.88E-05	1.61E-05	CYP1A2/MSMO1/SQLE/FDFT1/CYP39A1/CYP7B1/MVD/CYP7A1/PON1/GBA/NSDHL/CYP4V2	12
GO:0008203	GO:0008203	cholesterol metabolic process	11/149	149/18862	2.80E-08	3.38E-05	2.89E-05	CYP1A2/MSMO1/SQLE/FDFT1/CYP39A1/CYP7B1/MVD/CYP7A1/PON1/GBA/NSDHL	11
GO:1902652	GO:1902652	secondary alcohol metabolic process	11/149	158/18862	5.13E-08	3.38E-05	2.89E-05	CYP1A2/MSMO1/SQLE/FDFT1/CYP39A1/CYP7B1/MVD/CYP7A1/PON1/GBA/NSDHL	11
GO:0008202	GO:0008202	steroid metabolic process	15/149	329/18862	5.44E-08	3.38E-05	2.89E-05	CYP1A2/MSMO1/SQLE/LGMN/HSD17B2/FDFT1/CYP39A1/CYP7B1/MVD/CYP7A1/PON1/GBA/NSDHL/TSPO/CYP4V2	15
GO:0034976	GO:0034976	response to endoplasmic reticulum stress	14/149	296/18862	9.85E-08	4.90E-05	4.19E-05	ERP44/HYOU1/PDIA4/MANF/HSPA5/TPP1/DNAJC3/SDF2L1/DNAJB11/UBQLN1/HSP90B1/HSPA1A/UBXN1/HM13	14
GO:0035966	GO:0035966	response to topologically incorrect protein	11/149	206/18862	7.48E-07	0.00030982	0.00026484	ERP44/HSPH1/HYOU1/MANF/HSPA5/TPP1/DNAJC3/SDF2L1/DNAJB11/HSP90B1/HSPA1A	11
GO:0006986	GO:0006986	response to unfolded protein	10/149	185/18862	2.17E-06	0.00076211	0.00065146	ERP44/HSPH1/HYOU1/MANF/HSPA5/TPP1/DNAJC3/DNAJB11/HSP90B1/HSPA1A	10
GO:0006457	GO:0006457	protein folding	11/149	233/18862	2.50E-06	0.00076211	0.00065146	ERP44/HSPH1/PDIA4/PPIB/HSPA5/DNAJC3/SDF2L1/DNAJB11/HSP90B1/HSPA1A/UBXN1	11
GO:0006694	GO:0006694	steroid biosynthetic process	10/149	190/18862	2.76E-06	0.00076211	0.00065146	MSMO1/SQLE/HSD17B2/FDFT1/CYP39A1/CYP7B1/MVD/CYP7A1/NSDHL/TSPO	10
GO:0061077	GO:0061077	chaperone-mediated protein folding	6/149	63/18862	1.03E-05	0.0025594	0.00218781	HSPH1/PDIA4/PPIB/HSPA5/SDF2L1/HSPA1A	6

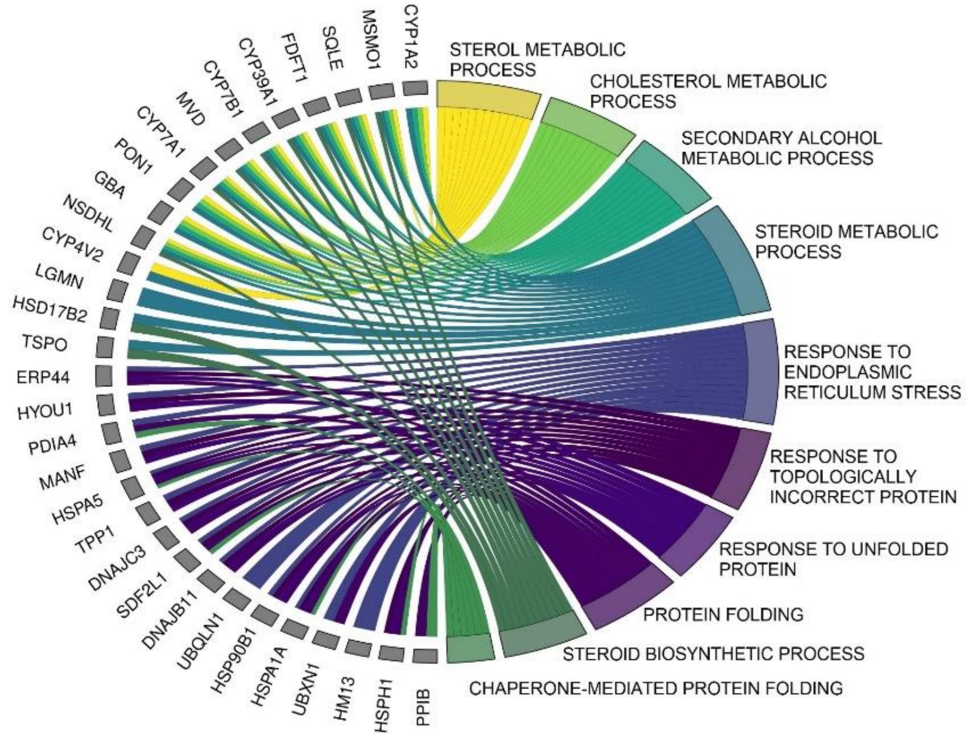

Supplementary Table S5: Gene Ontology biological process enrichment analysis of downregulated proteins.

183 proteins that were found significantly downregulated in the livers of miR-22KO mice at 3 months of HFD (Gjorgjieva et al.,JPM.2022) were submitted to an Gene ontology enrichment analysis of biological process *via* the R software packages GOdb and clusterProfiler. The 10 most significantly enriched processes were retained and presented as a chordplot.

	Gene	Predicted in mouse	Validated in mouse	Predicted in human	Validated in human	Metabolic gene	Function
1	**ENO1**	Yes	No	Yes	Yes PMID: 29913409	Yes	Involved in glycolysis and AMPK/mTOR signaling PMID: 34671213
2	ACAT2	No	No	No	No		
3	TYMP	No	No	No	No		
4	**HPGD**	No	No	Yes	No	No	Prostaglandin degradation, migration PMID: 22072156
5	**LGALS1**	Yes	No	Yes	Yes, in HCC PMID 27494859	No	Cell migration, angiogenesis, ECM-cell interactions and signaling PMID: 20200618
6	ABCC3	No	No	No	No		
7	**THBS1**	Yes	No	No	No	No	Regulation of angiogenesis and TGFβ signaling PMID: 27492250
8	UGT1A9	No	No	No	No		
9	**GSTM3**	Yes	No	No	No	No	Regulation of glutathione levels and detoxification PMID: 29774096
10	ADH4	No	No	No	No		
11	**GCLC**	Yes	No	Yes	No	No	Regulation of glutathione levels PMID: 16081425
12	ALB	No	No	No	No		
13	AGMO	No	No	No	No		
14	**PLIN2**	Yes	No	No	No	Yes	Regulation of lipid synthesis and composition PMID: 27679530
15	ACSS3	No	No	No	No		
16	GAPDH	No	No	No	No		
17	**PKLR**	No	No	Yes	No	Yes	Regulation of glucose uptake and mitochondrial activity in HCC PMID: 30615941
18	GSTA1	No	No	No	No		
19	ALDOB	No	No	No	No		
20	CYP17A1	No	No	No	No		
21	**FABP1**	Yes	No	No	No	Yes	Fatty acid uptake PMID: 26443794
22	**PIGR**	No	No	Yes	No	No	Ribosome pathway upregulation in HCC PMID: 33390805

Supplementary Table S6: Identification of miR-22 targets involved in hepatocarcinogenesis.

The gene names of 148 proteins that were found significantly upregulated in the livers of miR-22KO mice at 12 weeks of HFD (Gjorgjieva et al.,JPM.2022) were transformed from mouse gene to a human gene name *via* the The Database for Annotation, Visualization and Integrated Discovery (DAVID) (https://david.ncifcrf.gov/). The list of human genes obtained through DAVID contained 127 candidates, as 21 genes from the mouse list did not have a specific ortholog in human. These 127 genes were further cross-referenced with a list of genes associated to HCC from the Metacore database and we thus obtained 22 candidates of miR-22 targets associated to HCC (Fig.4B). Finally, these 22 candidates were screened whether they are a predicted/validated human/mouse target of miR-22 *via* the miRWalk database (http://mirwalk.umm.uni-heidelberg.de/ predicted by at least 3 software). 10 out of 22 candidates were found to be predicted/validated targets of miR-22, among which 4 were considered as metabolic targets (yellow) and 6 were considered as non-metabolic targets (green).
